# Regulation of autophagy and the ubiquitin–proteasome system by the FoxO transcriptional network during muscle atrophy

**DOI:** 10.1038/ncomms7670

**Published:** 2015-04-10

**Authors:** Giulia Milan, Vanina Romanello, Francesca Pescatore, Andrea Armani, Ji-Hye Paik, Laura Frasson, Anke Seydel, Jinghui Zhao, Reimar Abraham, Alfred L. Goldberg, Bert Blaauw, Ronald A. DePinho, Marco Sandri

**Affiliations:** 1 Venetian Institute of Molecular Medicine, via Orus 2, 35129 Padova, Italy; 2 Department of Biomedical Sciences, University of Padova, 35131 Padova, Italy; 3 Department of Pathology and Laboratory Medicine, Weill Cornell Medical College, New York, New York 10065, USA; 4 Department of Cell Biology, Harvard Medical School, Boston, Massachusetts 02115, USA; 5 Department of Cancer Biology, U3 Pharma GmbH, Martinsried 82152, Germany; 6 Department of Cancer Biology, University of Texas MD Anderson Cancer Center, Houston, Texas 77030, USA; 7 Institute of Neuroscience, Consiglio Nazionale delle Ricerche, 35129 Padova, Italy; 8 Department of Medicine, McGill University, Montreal, Quebec H3AoG4, Canada; 9 Dulbecco Telethon Institute at Telethon Institute of Genetics and Medicine (TIGEM), 80131 Napoli, Italy

## Abstract

Stresses like low nutrients, systemic inflammation, cancer or infections provoke a catabolic state characterized by enhanced muscle proteolysis and amino acid release to sustain liver gluconeogenesis and tissue protein synthesis. These conditions activate the family of Forkhead Box (Fox) O transcription factors. Here we report that muscle-specific deletion of FoxO members protects from muscle loss as a result of the role of FoxOs in the induction of autophagy–lysosome and ubiquitin–proteasome systems. Notably, in the setting of low nutrient signalling, we demonstrate that FoxOs are required for Akt activity but not for mTOR signalling. FoxOs control several stress–response pathways such as the unfolded protein response, ROS detoxification, DNA repair and translation. Finally, we identify FoxO-dependent ubiquitin ligases including MUSA1 and a previously uncharacterised ligase termed SMART (Specific of Muscle Atrophy and Regulated by Transcription). Our findings underscore the central function of FoxOs in coordinating a variety of stress-response genes during catabolic conditions.

## Abstract (web summary)

FoxO transcription factors promote muscle atrophy in response to stresses such as low nutrient availability. By generating muscle-specific FoxO triple-knockout mice, Milan *et al.* identify mechanisms by which the FoxO transcriptional network coordinates autophagic and proteasomal protein degradation.

## Graphical abstract

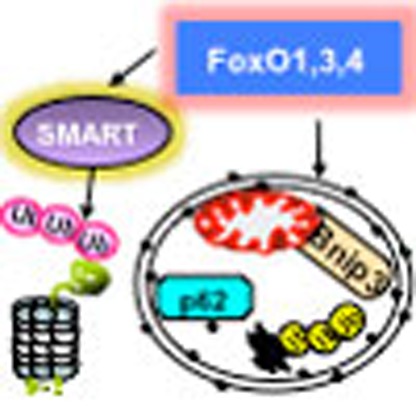

Environmental transformations have selected for species that are able to quickly and efficiently regulate their physiology. Under stress conditions, mammalian cells activate compensatory mechanisms to adapt themselves to new situations. Depending on the stimuli, the adaptive response either require minor and fast metabolic changes or involves major and sustained adjustments that need transcription-dependent adaptations. Muscles are the largest protein reservoir in the body and serve as a source of amino acids that can be used for energy production by various vital organs (including heart, liver and brain) during catabolic periods, such as in cancer, sepsis, burn injury, heart failure and AIDS^1^. However, excessive and sustained protein degradation in skeletal muscle, and the resulting muscle loss (cachexia), is highly detrimental and can lead to death. Moreover, excessive loss of muscle mass is a poor prognostic index and impairs the efficacy of many different therapeutic treatments. Thus, cachexia ultimately aggravates diseases and increases morbidity and mortality.

In eukaryotic cells, most proteins are degraded via two proteolytic systems: the ubiquitin–proteasome and the autophagy–lysosome. In skeletal and cardiac muscles the two systems are coordinately regulated to remove proteins and organelles in atrophying cells^2^,^3^. Muscle atrophy requires a transcription-dependent programme to regulate a group of genes that are commonly up or downregulated in atrophying muscles during different catabolic conditions and that are named atrophy-related genes or atrogenes^1^,^4^,^5^,^6^,^7^,^8^. These genes encode enzymes that catalyse important steps in autophagy–lysosome, ubiquitin–proteasome, unfolded protein response, ROS detoxification, DNA repair, mitochondrial function and energy balance pathways. The transcription factors that orchestrate this complex gene network have been the focus of active investigation.

The two atrogenes with the greatest induction are two muscle-specific ubiquitin ligases, namely *atrogin1/MAFbx* and *MuRF1*^4^,^5^. Mice lacking these two enzymes are partially resistant to muscle atrophy induced by denervation^4^. However, the action of these two ubiquitin ligases cannot account for the degradation of all muscle proteins. A number of additional unknown ubiquitin ligases (E3s) are presumably activated during atrophy to promote the clearance of myofibrillar and soluble proteins and/or to limit anabolic processes. We have recently established that these atrophy-related ubiquitin ligases, as well as protein breakdown in general, are blocked by the growth-promoting IGF1/AKT pathway^8^,^9^. Members of the Forkhead Box (Fox) O family (FoxO1, 3 and 4), downstream targets of AKT, were identified as the main transcription factors regulating atrogin1 expression^8^. Importantly, we have also found that FoxO3 regulates autophagy coordinating the proteasomal-dependent removal of proteins with the autophagy-dependent clearance of organelles^2^,^10^. The use of FoxO1 knockout mice has established a role in muscle protein homeostasis showing partial protection from muscle loss during chronic kidney disease^11^. FoxO1 deficiency was associated with partial reduction in the expression of atrogin1, MuRF1 and the lysosomal enzyme, Cathepsin-L^11^,^12^. These studies linking FoxOs and muscle wasting prompted us to explore more fully the specific function of the entire FoxO family in skeletal muscle maintenance/loss and the potential for functional redundancy among the FoxO members and to illuminate the atrogene expression network focused on the identification of novel FoxO-dependent atrophy-related genes.

Here we show that specific deletion of FoxOs in skeletal muscles prevents muscle loss and force decline in response to fasting and denervation because the FoxO family is required for the induction of several atrophy-related genes. Moreover, we identify a novel ubiquitin ligase, named SMART (Specific of Muscle Atrophy and Regulated by Transcription), that plays a critical role in protein ubiquitination after denervation.

## Results

### Generation of a triple FoxO1,3,4 muscle-specific knockout

**Figure 1:**
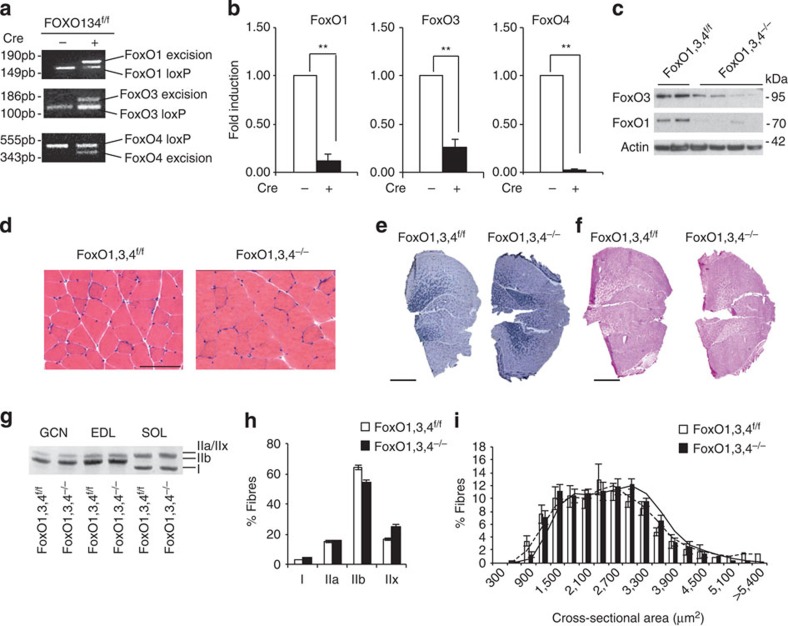
Deletion of FoxOs is permissive for normal muscle function. (**a**) PCR analysis with genomic DNA from FoxO1,3,4^f/f^ and FoxO1,3,4^−/−^ gastrocnemius muscles. (**b**) *FoxO1*, *FoxO3* and *FoxO4* mRNA expression were quantified by RT–PCR in Tibialis Anterior (TA) muscle of *FoxO1,3,4*^*−/−*^ and control mice. *n*=4 each group. (**c**) Immunoblot showing reduction of FoxO1 and FoxO3 proteins in homogenates of *FoxO1,3,4*^*−/−*^ gastrocnemius muscles. Data are representative of three independent experiments. (**d**) Haematoxylin and eosin (Scale bar, 100 μm), (**e**) SDH (Scale bar, 1 mm) and (**f**) PAS staining (Scale bar, 1 mm) showing normal morphology, fibre type and glycogen of *FoxO1,3,4*^*−/−*^ gastrocnemius muscle. (**g**) SDS–PAGE and (**h**) immunohistochemistry analysis of myosin heavy chain type I, IIA, IIB and IIX proteins in gastrocnemius muscles showing no differences between *FoxO1,3,4*^*−/−*^ and *FoxO1,3,4*^*f/f*^ mice. Data are representative of three independent experiments. (**i**) Frequency histograms showing the distribution of cross-sectional areas (μm^2^) in TA of *FoxO1,3,4*^*f/f*^ (white bars) and *FoxO1,3,4*^*−/−*^ (black bars) fibres, *n*=4, each group. Data are shown as mean±s.e.m. Error bars indicate s.e.m. **P<0.01 (Student's *t*-test).

Since muscle wasting requires a transcriptional-dependent programme to induce the expression of a subset of genes and because FoxO family is sufficient to induce muscle atrophy, we generated mice with adult skeletal muscle-specific deletion of all three FoxO family members to unravel the role of this family in gene regulation and muscle adaptation during catabolic conditions. To that end, *FoxO1/3/4*-floxed mice (*FoxO1/3/,4*^*f/f*^) were crossed with a transgenic line expressing Cre recombinase under the control of muscle-specific MLC1f promoter (hereafter referred to as *FoxO1,3,4*^*−/−*^). PCR analysis confirmed deletion of the floxed sequence from genomic DNA extracted from skeletal muscle (Fig. 1a). Quantitative reverse transcription–PCR (RT–PCR) and Western blot analyses showed a significant decrease or elimination of *FoxO1*, *FoxO3* and *FoxO4* transcripts and proteins, respectively (Fig. 1b,c). Since FoxO4 is mainly expressed in striated muscles^13^, while FoxO1 and 3 are expressed in a variety of tissues, the traces of *FoxO1* and *3* messenger RNAs (mRNAs) come from endothelial, fibroblasts, macrophages and blood cells. Thus, we confirm a genetic model of muscle-specific inhibition of FoxO1,3,4 family.

### Triple FoxO1,3,4 deletion in muscle does not affect fibre type

*FoxO1,3,4*^*−/−*^ mice were indistinguishable in gross appearance from age-matched control *FoxO1,3,4*^*f/f*^ mice and histological analysis of adult muscles revealed normal muscle architecture and absence of myopathic features such as centrally nucleated fibres (Fig. 1d). Succinate dehydrogenase staining showed no major changes in distribution of small β-oxidative mitochondrial rich versus large glycolytic mitochondrial poor fibres (Fig. 1e). Since FoxOs are important for glucose homeostasis in liver, we monitored glycogen levels in muscle. PAS staining revealed an almost identical distribution of glycogen stores (Fig. 1f). Analyses of myosin heavy chain expression (Fig. 1g) and distribution (Fig. 1h) did not reveal any significant difference between wild-type and FoxO1,3,4 knockout mice. These findings support the view that FoxO deficiency does not affect fibre type determination and basal glucose homeostasis and is permissive for normal muscle function. Quantification of cross-sectional area of fast muscles (Tibialis Anterior (TA) and Gastrocnemius) did not show any significant difference between knockout and controls (Fig. 1i, Supplementary Fig. 1).

### FoxO inhibition prevents muscle loss and weakness

**Figure 2:**
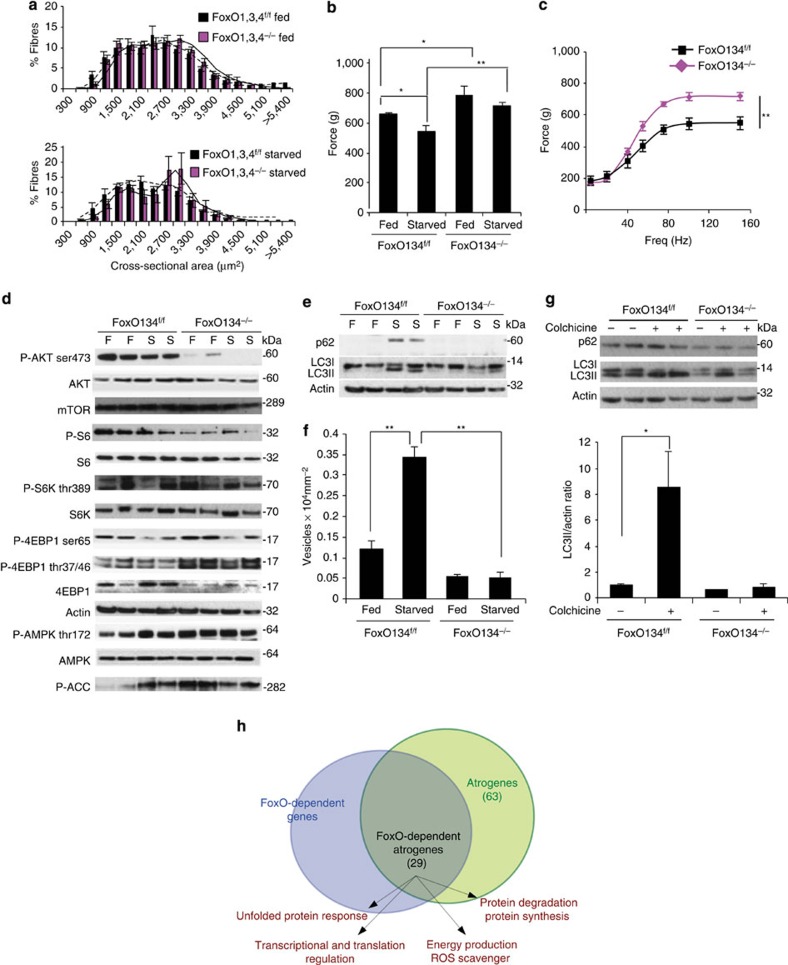
Deletion of FoxOs prevents muscle loss and weakness during fasting. (**a**) Frequency histograms of cross-sectional areas (μm^2^) of *FoxO1,3,4*^*f/f*^ (black bars) and *FoxO1,3,4*^*−/−*^ (magenta bars) fibres in fed (upper panel) and fasted (lower panel) conditions, *n*=4, each group. (**b**) Force measurements preformed *in vivo* on gastrocnemius showed that *FoxO1,3,4*^*−/−*^ muscles preserve maximal tetanic force after fasting; *n*=6 muscles in each group. (**c**) Force/frequency curve of starved gastrocnemius muscle underlines the important protection achieved by the absence of FoxOs; *n*=6 muscles in each group. (**d**) Immunoblot of protein extracts from gastrocnemius muscles. Phosphorylation of AKT and S6 is reduced in fed and starved *FoxO1,3,4*^*−/−*^ muscles when compared with controls. Data are representative of three independent experiments. (**e**) Immunoblot analysis of p62 and LC3 in homogenates of gastrocnemius muscles from fed and starved *FoxO1,3,4*^*−/−*^ or controls. Fasting did not induce LC3 lipidation and p62 upregulation in FoxO-deficient muscles. Data are representative of three independent experiments. (**f**) Quantification of GFP–LC3-positive vesicles in *FoxO1,3,4*^*f/f*^ and *FoxO1,3,4*^*−/−*^ TA muscles; *n*=4 muscles in each group (**g**) Autophagy flux is not increased in FoxO-deficient TA muscles. Inhibition of autophagy–lysosome fusion by colchicine treatment induces accumulation of LC3II band in starved control but not in starved *FoxO1,3,4*^*−/−*^ muscles. Upper panel: immunoblot analysis of gastrocnemius homogenates. Lower panel: quantification of LC3 lipidation. *n*=4 muscles in each group (**h**) The scheme shows the overlap between FoxO-dependent genes, identified by gene expression profiling of fed (*n*=4) and fasted (*n*=4) muscles from *FoxO1,3,4*^*f/f*^ and *FoxO1,3,4*^*−/−*^ and atrophy-related genes or atrogenes. The data in the graphs are shown as mean±s.e.m. Error bars indicate s.e.m. *P<0.05, **P<0.01 (Student's t-test).

To further characterize the role of FoxO1,3,4 in skeletal muscles, we then analysed the phenotype of *FoxO1,3,4*^*−/−*^ mice under conditions of muscle wasting. Initially, we used fasting as a model of muscle loss since it is an established condition that induces nuclear translocation of FoxO members and their binding to target promoters^2^,^8^,^14^ (Supplementary Fig. 2) and we compared FoxO1,3,4 null muscles with controls. Importantly, FoxO1,3,4 knockout mice were completely spared from muscle loss after fasting (Fig. 2a, Supplementary Fig. 3). To understand whether sparing of muscle mass is also functionally relevant, we measured muscle force in living animals. While control fasted animals became significantly weaker than fed ones, *FoxO1,3,4*^*−/−*^ gastrocnemius muscles did not loose strength after fasting (Fig. 2b). Importantly the comparison of force/frequency curve of fasted wild-type versus fasted FoxO1,3,4 null muscle underlined the important protection achieved by the absence of FoxO family when nutrients are low or absent (Fig. 2c). These findings confirm that the absence of FoxO members prevents atrophy and profound weakening. To explain this profound effect on sparing force and muscle mass, we monitored the level of the contractile protein, myosin, when nutrients are removed. As expected, fasting induced an important reduction of myosin content in controls, while FoxO1,3,4 knockout were completely protected (Supplementary Fig. 4a). The maintenance of myosins in knockout is consequent to inhibition of protein ubiquitination (Supplementary Fig. 4b-c).

### FoxOs are critical for autophagy and protein ubiquitination

To explore the basis for sparing of muscle mass in absence of nutrients, we audited the signalling pathways linked to Insulin/IGF1 and energy. Interestingly, Akt, S6K and S6 phosphorylation, both in fed and fasted muscles, were reduced in knockout mice, while phosphorylation of 4EBP1, the other mTORC1 downstream target, was increased in FoxO-deficient TA muscle (Fig. 2d, Supplementary Fig. 5a). The energy–stress sensor AMPK and its downstream target ACC did not significantly differ from controls in fed and fasting conditions (Fig. 2d). Therefore, the changes in the pathways downstream energy and insulin/nutrients do not match with the important sparing of muscle mass in knockout mice when nutrients are absent.

Since the absence of nutrients is a potent stimulus for autophagy-dependent degradation and since FoxO is an inducer, while mTOR is a suppressor of autophagy–lysosome system, we checked the status of this system in *FoxO1,3,4*^*−/−*^ mice. We monitored LC3 lipidation, LC3-positive vesicles and autophagy flux in *FoxO1,3,4*^*f/f*^ and *FoxO1,3,4*^*−/−*^ mice. Fasting induced LC3 lipidation (Fig. 2e, Supplementary Fig. 5b) and vesicles formation (Fig. 2f) in control but not in FoxO-deficient muscles. Autophagy flux was monitored by treating animals with colchicine, an established inhibitor of microtubule-mediated delivery of autophagosome to lysosome^15^. The inhibition of autophagosome fusion to lysosomes led to accumulation of lipidated LC3 in controls but not in FoxO1,3,4-deficient muscles (Fig. 2g). In conclusion, during fasting, despite mTOR signalling being partially inhibited, autophagy enhancement is completely blocked in *FoxO1,3,4*^*−/−*^ muscle.

Absence of nutrients activates also the ubiquitin–proteasome system, leading to an increase of protein ubiquitination and, consequently, in proteasome-dependent degradation. Fasting resulted in an increase in both lysine-48 and lysine-63 poly-ubiquitinated proteins (Supplementary Fig. 4b,c), confirming an activation of the ubiquitination process in controls. Importantly, this increase was totally abolished in FoxO1,3,4 knockout mice.

### Half of the atrophy-related genes are under FoxO regulation

**Figure 3:**
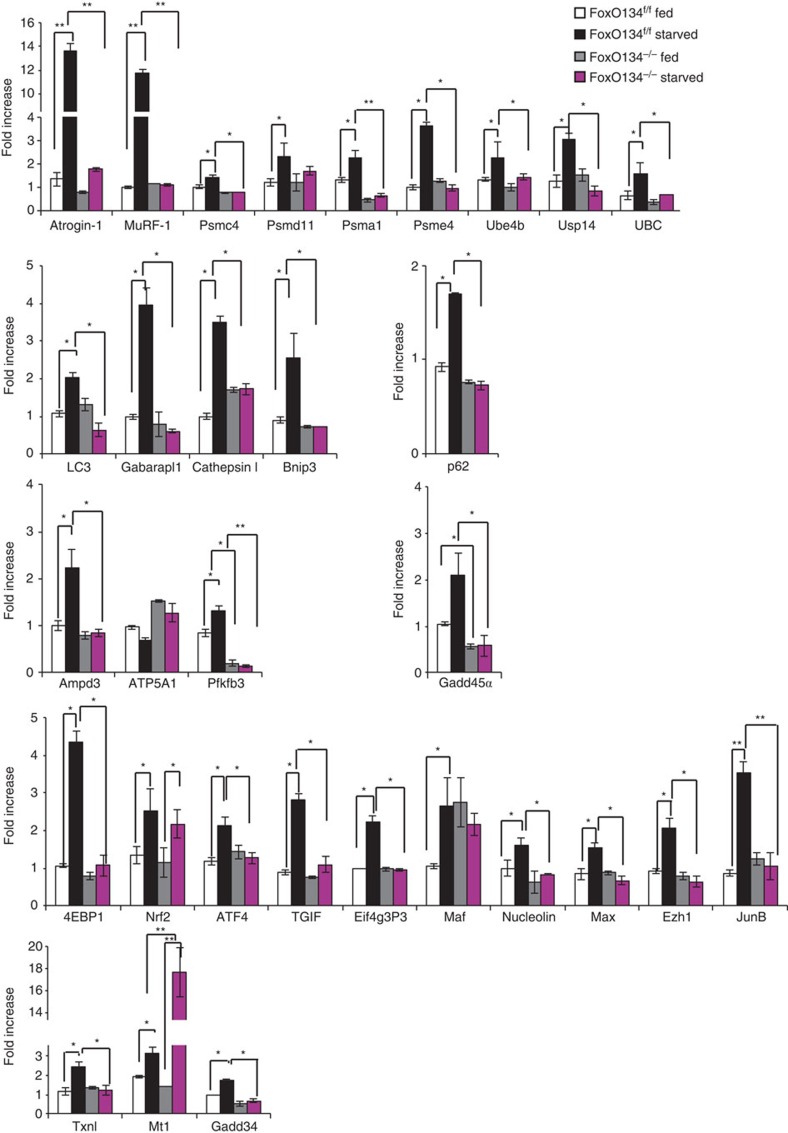
Absence of FoxOs prevent the induction of critical atrogenes. Quantitative RT–PCR of atrogenes from fed and 24-h starved tibialis anterior of control and *FoxO1,3,4*^*−/−*^ mice. Data are normalized to GAPDH and expressed as fold increase of control-fed animals. *n*=4 muscles in each group Values are mean±s.e.m. *P<0.05,**P<0.01. (Student's *t*-test).

Since muscle atrophy is characterized by transcription-dependent regulation of atrogenes (atrophy-related genes) and FoxO deficiency protects from muscle loss, we sought to identify genes under FoxO regulation. Gene expression profiles of fed and fasted control and *FoxO1,3,4*^*−/−*^ muscles were compared with identify genes with blunted induction in the fasted *FoxO1,3,4*^*−/−*^ mice relative to controls. Cross-referencing with the list of known atrogenes^7^, these analyses revealed that 29 of the 63 atrophy-related genes require FoxO for their normal induction during fasting (Fig. 2h, Supplementary Fig. 10). Quantitative RT–PCR confirmed that 26 of the atrophy-related genes were not induced in FoxO null muscles during fasting (Fig. 3). Consistent with morphology and force measurements, the induction of genes involved in protein breakdown was completely blocked in knockout mice. In fact both the ubiquitin system including the ubiquitin ligases (*Atrogin1* and *MuRF1*), the ubiquitin gene (*UBC*), the de-ubiquitinating enzyme (*USP14*), the E3/E4 enzyme (*Ube4b*), several proteasome subunits (*Psme4/PA200, Psma1, Psmc4/Rpt3* and *Psmd11/Rpn6*) as well as the autophagy-related genes (*LC3, Gabarapl, Bnip3, Cathepsin L* and *p62/SQSTM1*) were completely blocked in *FoxO1,3,4*^*−/−*^ mice. Other pathways that are inhibited in knockout muscles are related to the unfolded protein response (*ATF4* and *GADD34*), protein synthesis (*4EBP1* and *eIF4g*), transcription regulators that negatively control Smad2/3 (*TGIF*) or positively affect Myc (*MAX*), DNA repair/chromatin remodelling (*GADD45α*) and ribosome transcription/maturation/assembly (*Nucleolin*). Expression of genes that are related to oxidative stress were more variable; *Nrf2/Nfe2l2* was not affected by FoxO deletion, while *Thioredoxin* was suppressed and *Metallothionein1* was significantly induced in the absence of FoxO factors.

Western blot analyses of some atrogenes involved in autophagy (p62, Gabarapl and Bnip3), chromatin remodelling/DNA repair (GADD45α), protein synthesis (4EBP1) and ubiquitin–proteasome confirmed their upregulation during fasting in control animals but not in FoxO1,3,4 knockout (Supplementary Fig. 6).

### Inducible FoxOs loss phenocopies the conditional knockout

**Figure 4:**
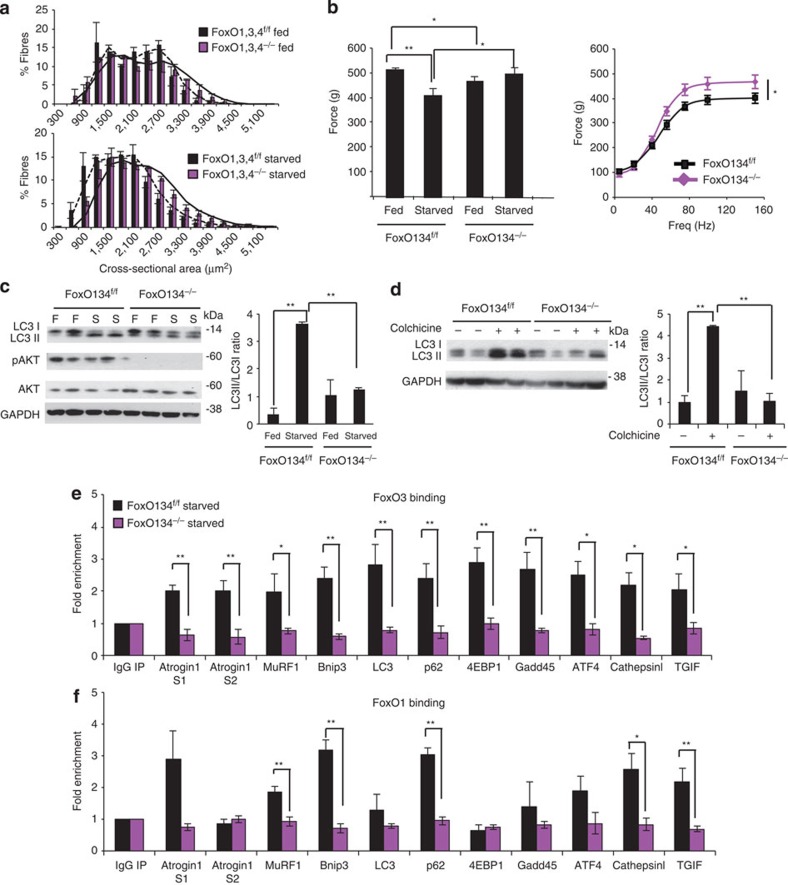
Acute inhibition of FoxOs phenocopies the conditional FoxO1,3,4 knockout. (**a**) Frequency histograms of gastrocnemius muscles showing the distribution of cross-sectional areas (μm^2^) of inducible muscle-specific FoxO1,3,4 mice after tamoxifen-dependent deletion of *FoxO1,3,4* genes (*FoxO1,3,4*^*f/f*^: black bars and *FoxO1,3,4*^*−/−*^: magenta bars) in fed (upper panel) and fasted (lower panel) conditions, *n*=3, each group. (**b**) Force measurements preformed *in vivo* on gastrocnemius muscle showed that acute inhibition of FoxOs in adulthood prevents force drop during fasting. *n*=6 muscles in each group. Freq: Frequency. (**c**) Left, immunoblotting analyses of gastrocnemius homogenates after acute deletion of FoxO1,3,4^*−/−*^ and controls. Right, quantification of LC3 lipidation. Data are representative of three independent experiments. (**d**) Autophagy flux is not increased in FoxO-deficient TA muscles. Inhibition of autophagy–lysosome fusion by colchicine treatment induces accumulation of LC3II band in starved control but not in starved *FoxO1,3,4*^*−/−*^ muscles. Left, immunoblotting analyses of gastrocnemius homogenates. Right, quantification of LC3 lipidation. (**e**,**f**) ChIP quantitative RT–PCR shows the recruitment of FoxO3 and FoxO1 on the promoters of selected atrophy-related genes. ChIP assays were performed in starved control and *FoxO1,3,4*^*−/−*^ TA muscles. IgG was used as the reference. *n*=3 for each group. Data are shown as mean±s.e.m. Error bars indicate s.e.m. *P<0.05, **P<0.01 (Student's *t*-test). S1: FoxO binding site 1; S2: FoxO binding site 2.

To corroborate our findings, we also explored the impact of somatic deletion of FoxO in the adult via a tamoxifen-inducible muscle-specific FoxO knockout model. Following documentation of tamoxifen-induced deletion of FoxOs (Supplementary Fig. 7), we assessed muscle loss during fasting by monitoring muscle mass and muscle force. Similar to the above, we found that somatic deletion of FoxO1,3,4 prevented muscle loss (Fig. 4a) and muscle weakness during fasting (Fig. 4b). Consistent with the conditional knockout, Akt activation and LC3 lipidation was reduced in FoxO knockout when compared with controls (Fig. 4c). Autophagy flux measurements confirmed that FoxOs are required for autophagy induction (Fig. 4d). We then tested whether inhibition of FoxO members in adulthood blocked the activation of the atrogenes. Indeed, we confirmed most of the data obtained by the conditional FoxO1,3,4 knockout (Supplementary Fig. 8). The fact that two different FoxO1,3,4 knockout mice resulted in comparable biological effects strongly supports the conclusion that the FoxO family is a master regulator of the atrophy programme under low nutrient conditions.

### FoxOs directly regulate atrophy-related genes

To determine whether the regulation of these atrogenes requires direct binding of FoxO transcription factors to their promoters and to identify the binding sites we performed chromatin immunoprecipitation (ChIP) experiments on fasted muscles of *FoxO1,3,4*^*f/f*^. We also used *FoxO1,3,4*^*−/−*^ as a negative control to validate the specificity of the immunoprecipitation. FoxO3 was recruited to almost all of the promoters so far analysed (Fig. 4e). We then asked whether the activation of these genes is strictly dependent on FoxO3 or whether other FoxO members can bind the same promoter region. We could immunoprecipitate FoxO1 but not FoxO4 because we did not find any ChIP grade antibody that was specific for FoxO4 without crossreacting with other FoxOs. Interestingly, FoxO1 was found to be significantly recruited to some promoters such as *MuRF1*, *p62*, *Cathepsin L* and *TGIF* (Fig. 4f). This finding suggests that regulation of some genes is shared by different FoxO members, while others preferentially recruit FoxO3.

### FoxOs are required for denervation-dependent atrophy

**Figure 5:**
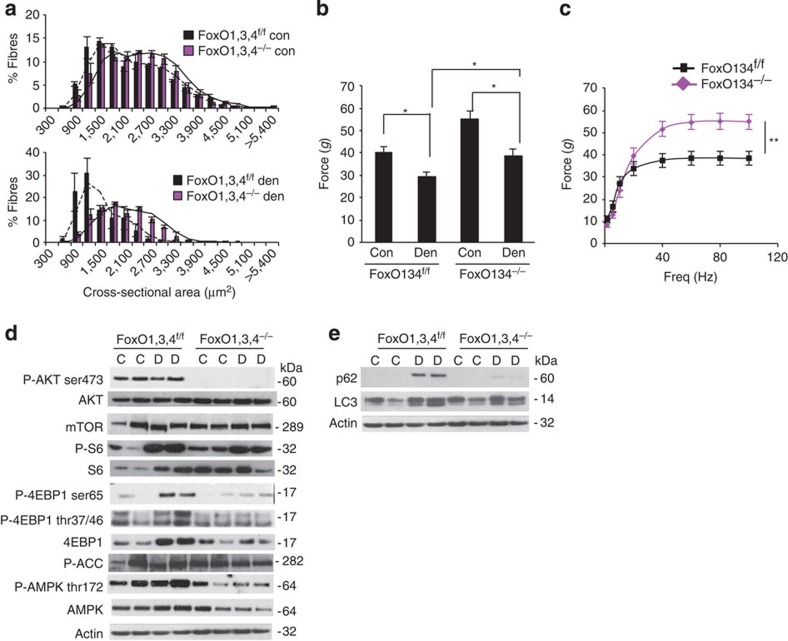
Deletion of FoxOs in skeletal muscle partially prevents atrophy during denervation. (**a**) Frequency histograms of gastrocnemius muscles from *FoxO1,3,4*^*−/−*^ and control mice showing the distribution of cross-sectional areas (μm^2^) of *FoxO1,3,4*^*f/f*^ (black bars) and *FoxO1,3,4*^*−/−*^ (magenta bars) in control (upper panel) or in denervation (lower panel). *n*=4 muscles each groups. (**b**) Force measurements preformed *ex vivo* on soleus muscles show that *FoxO1,3,4*^*−/−*^ muscles are stronger than controls, both in basal condition and after 14 days from denervation. *n*=6 muscles in each group. (**c**) Force/frequency curves of denervated soleus highlight the higher strength generated by *FoxO1,3,4*^*−/−*^ muscles when compared with controls. *n*=6 muscles in each group. (**d**) Immunoblots of gastrocnemius protein extracts reveal a decrease of AKT phosphorylation both in contralateral and in denervated muscles of *FoxO1,3,4*^*−/−*^ mice. The increase of 4EBP1 protein is blunted in FoxOs knockout mice. (**e**) Immunoblots of autophagy-related proteins. FoxOs are required for p62 induction, while LC3 is less lipidated after 3 days of denervation. Data are representative of three independent experiments. Data are shown as mean±s.e.m. Error bars indicate s.e.m. *P<0.05, **P<0.01 (Student's *t*-test). C, control; D, denervated.

**Figure 6:**
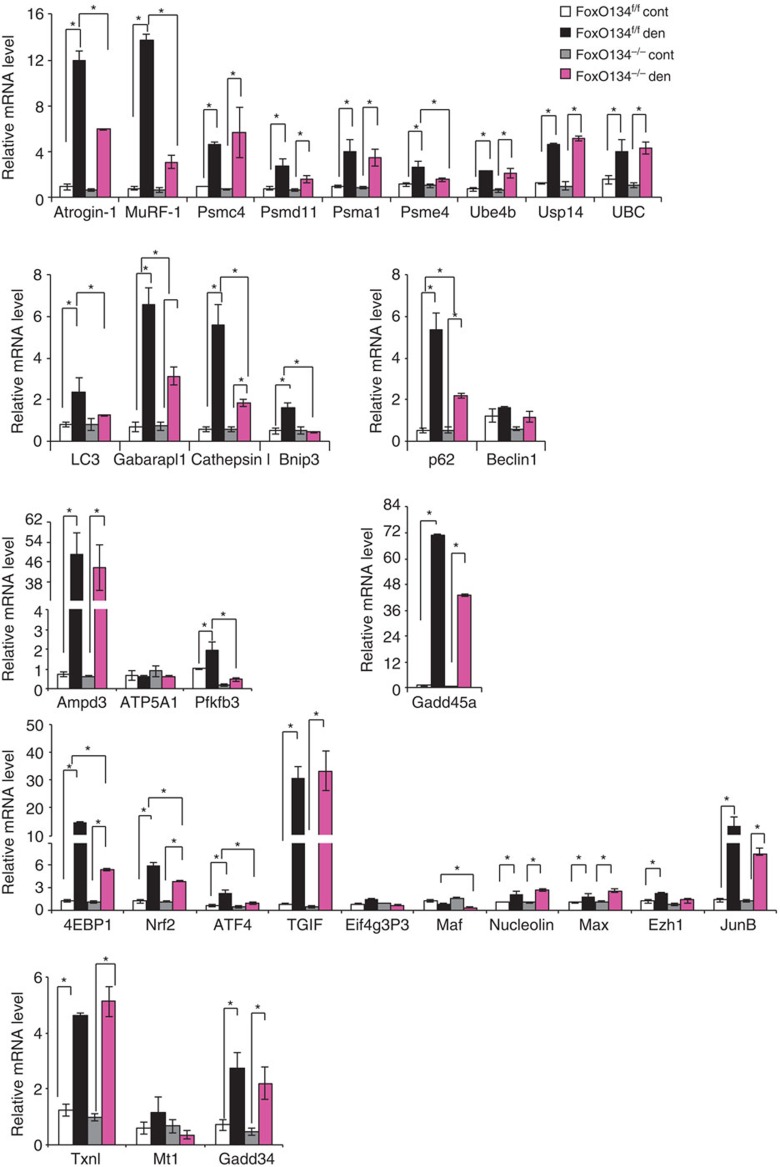
FoxOs are required for expression of several atrogenes after denervation. Quantitative RT–PCR of the indicated atrogenes after 3 days from denervation. Data are normalized to GAPDH and expressed as fold increase of control innervated muscles. Values are mean±s.e.m. *P<0,05, **P<0.01 (Student's *t*-test).; cont, control; den, denervated.

To further determine whether the role of FoxO1,3,4 is critical in different catabolic conditions, we then used denervation as another model of muscle atrophy. Quantification of fibre size revealed that FoxO-deficient muscles were partially protected from atrophy (Fig. 5a, Supplementary Fig. 9). When we monitored muscle force, we confirmed the histological data. Soleus muscle of FoxO1,3,4 knockout mice generated higher strength than controls in the basal condition (Fig. 5b) and FoxO deletion was able to partially prevent weakness and maintain the same force of innervated *FoxO1,3,4*^*f/f*^ (Fig. 5b,c). The decrease of force in denervated FoxO1,3,4-deficient soleus muscle is related to the presence of FoxO proteins in type I fibres that can promote protein breakdown in these myofibres. It is important to emphasize that the type I fibres express FoxOs because the promoter (MLC1f) driving the expression of Cre recombinase is active in type II fibres and not in type I fibres. However, comparison of the force/frequency curves of denervated wild-type versus denervated FoxO1,3,4 null soleus muscles underlines the important protection conferred by the absence of FoxO family when nerve is damaged (Fig. 5c). Analyses of signalling confirmed the downregulation of P-AKT in knockout muscles. Denervation induced an increase of total and phospho-4EBP1 protein in *FoxO1,3,4*^*f/f*^ but not in *FoxO1,3,4*^*−/−*^ muscle (Fig. 5d). The changes in the phosphorylation of 4EBP1 were not ascribed to activation of the cellular energy sensor AMPK, revealed by checking its phosphorylation level or the downstream target ACC. In contrast to fasting, the autophagy system was mildly affected by the absence of FoxOs. Indeed, LC3 lipidation was slightly decreased in *FoxO1,3,4*^*−/−*^ compared with controls after denervation (Fig. 5e). However, and consistent with fasting, the transcriptional upregulation of *p62/SQSTM1* in denervated muscles was greatly attenuated (Figs 5e and 6). When we tested the expression of the different atrophy-related genes, we found only a partial suppression of these genes (Fig. 6). Interestingly, the lists of FoxO-dependent genes during denervation and fasting do not completely overlap (Supplementary Fig. 10). For instance, the ROS detoxifying factor, *Nrf2/Nfe2l2*, was not affected by FoxO deletion in fasting, while it was less activated in denervated FoxO1,3,4 null muscles. Therefore, FoxO family members are necessary for muscle loss but their involvement in the atrophy programme depends on the catabolic condition.

### FoxO members are redundant

**Figure 7:**
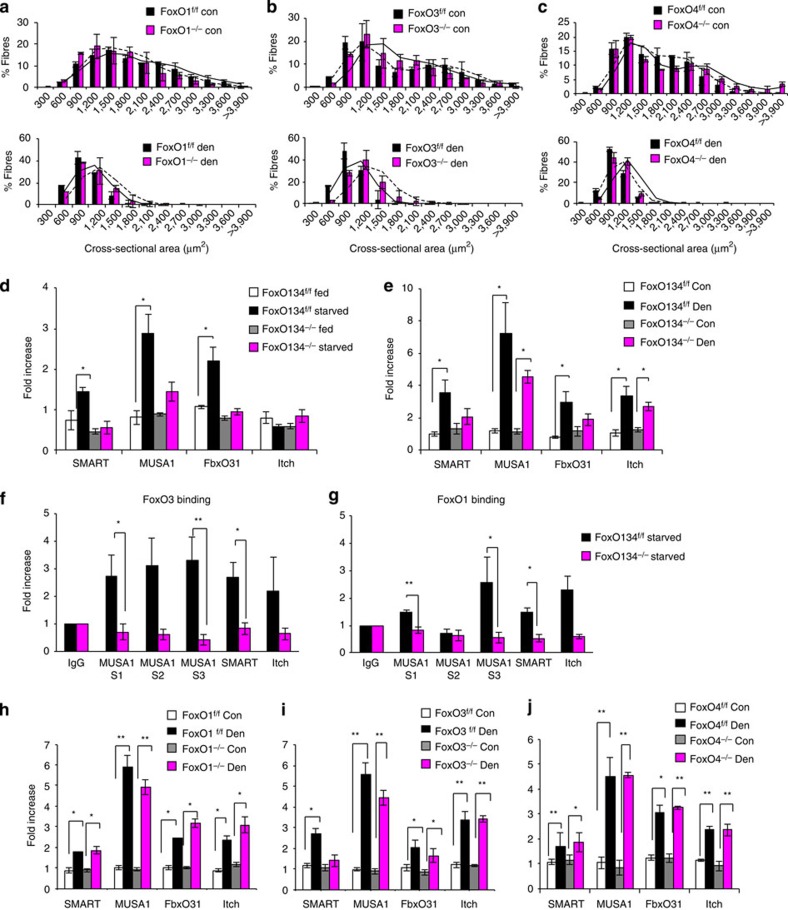
FoxO members are redundant and control a new set of ubiquitin ligases. (**a**–**c**) Frequency histograms showing the distribution of cross-sectional areas (μm^2^) of gastrocnemius muscles from muscle-specific (**a**) *FoxO1*^*−/−*^, (**b**) *FoxO3*^*−/−*^ and (**c**) *FoxO4*^*−/−*^ mice. Data are shown as mean±s.e.m. of four muscles each group. *P<0.05, **P<0.01. (**d**,**e**) qRT–PCR of the novel ubiquitin ligases *MUSA1, Fbxo21/SMART, Fbxo31*, *Itch* from 24 starved (**d**) or denervated (**e**) *FoxO1,3,4*^*f/f*^ and *FoxO1,3,4*^*−/−*^ mice. Data are normalized to GAPDH and expressed as fold increase of fed control mice. *n*=4 muscles for each group. (**f**) ChIP qPCR of FoxO3 and (**g**) FoxO1 on the promoters of *MUSA1*, *Fbxo21/SMART* and *Itch*. IgG was used as the reference. *n*=3. (**h**–**j**) qRT–PCR of *MUSA1, Fbxo21/SMART, Fbxo31, Itch* in (**h**) FoxO1, (**i**) FoxO3 and (**j**) FoxO4 knockout mice after 3 days of denervation. Values are normalized to GAPDH and expressed as fold increase of control mice. *n*=4 muscles for each group. Data are shown as mean±s.e.m. Error bars indicate s.e.m. *P<0.05, **P<0.01 (Student's *t*-test). con control; den, denervated. MUSA1 S1, FoxO-binding site1; MUSA1 S2, FoxO-binding site2; MUSA1 S3, FoxO-binding site3.

Since all FoxO members are expressed in muscles and are under AKT regulation, we investigated whether they play synergistic roles or have specific functions. To address this point, we generated muscle-specific individual FoxO knockout animals. We used denervation as a model of muscle atrophy. Deletion of FoxO1 did not protect from muscle atrophy (Fig. 7a, Supplementary Fig. 11). However the presence of FoxO1 is required for the optimal induction of several atrophy-related genes including *MuRF1, Cathepsin L, Gabarap L, GADD45a* and *TGIF* (Supplementary Fig. 12).

Inhibition of FoxO3 led to a small but significant protection from denervation-induced muscle atrophy (Fig. 7b). The protection was mainly achieved in oxidative fibres (Supplementary Fig. 13). Interestingly, when we checked the level of gene expression we found that *FoxO4* was strongly downregulated in *FoxO3*^*−/−*^ mice (Supplementary Fig. 14). Optimal expression of *Gabarapl, 4EBP1, Maf, Gadd45α, Pfkf3* and *Txn1* requires the presence of FoxO3 (Supplementary Fig. 14).

Ablation of FoxO4 did not significantly protect myofibres from atrophy after denervation (Fig. 7c and Supplementary Fig. 15) and did not reduce the expression of any atrogenes with the exception of a slight reduction of *GADD45α* and *Thioredoxin* (Supplementary Fig. 16).

### FoxOs regulate a novel set of ubiquitin ligases

To explain why inhibition of FoxO3 slightly reduced muscle atrophy despite the limited effects on induction of the atrophy-related genes, we looked for novel genes that might be involved in protein breakdown and that are selectively regulated by this FoxO member in denervated muscles. When we looked at the gene expression profiles in fasted muscles of FoxO1,3,4 knockout mice, we noticed a group of ubiquitin ligases that were upregulated in *FoxO1,3,4*^*f/f*^ but not in *FoxO1,3,4*^*−/−*^ mice. This set of ubiquitin ligases includes *MUSA1*, a novel E3, that we have recently found to be critical in muscle atrophy^16^, *Fbxo31*, an E3 of SCF family involved in cyclinD degradation and tumour suppression^17^, *Itch*, a HECT-type ubiquitin ligase that regulates the half-life of transcription factors such as JunB, c-Jun and p63^18^, and, finally, *Fbxo21*, a gene of unknown function but that contains an F-box motif and that we re-named *SMART*.

By quantitative RT–PCR, we could validate that upregulation of *MUSA1, SMART* and *FbxO31*, but not *Itch*, is blunted or partially blocked in fasted or denervated muscles of FoxO1,3,4, knockout mice (Fig. 7d,e). To further prove that the FoxO family is sufficient for their expression, we overexpressed FoxO3 in myotubes and we checked the expression of these E3s. Interestingly, FoxO3 was sufficient to induce the expression of *MUSA1* but not the other ubiquitin ligases (Supplementary Fig. 17). We then analysed the promoter regions of these genes and checked whether endogenous FoxO1 and FoxO3 directly bind these regions. ChIP experiments on fasted muscle confirmed that both FoxO1 and FoxO3 bind to the promoters of *MUSA1* and *SMART* (Fig. 7f,g). However, we did not find any significant recruitment of FoxOs on *Itch* and *Fbxo31* promoters (Fig. 7f,g).

We checked whether expression of these ubiquitin ligases was suppressed in denervated muscles of the single FoxO knockout mice. Interestingly, FoxO3 deletion completely blunted the induction of *SMART* while ablation of the other FoxO members did not elicit any effect on the upregulation of these ubiquitin ligases (Fig. 7h–j). Therefore, FoxO3 is the main regulator of SMART in denervated muscles and inhibition of SMART may explain the partial protection of FoxO3 null muscles after denervation (Fig. 7b and Supplementary Fig. 13).

### SMART is a novel E3 ligase required for muscle atrophy

**Figure 8:**
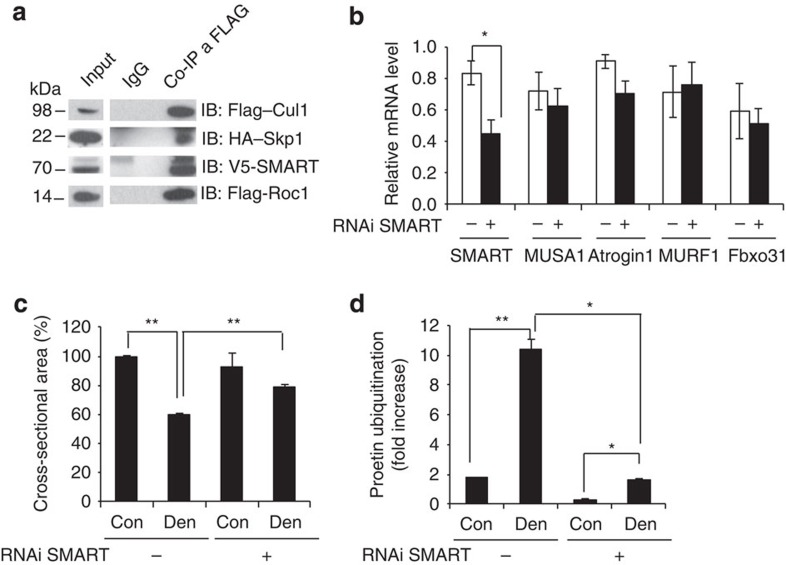
Smart is a novel ubiquitin ligase required for denervation-dependent atrophy. (**a**) Co-immunoprecipitation experiment showing that SMART is a F-box protein that forms a SCF complex. C2C12 muscle cell lines were transfected with SMART, Skp1, Cul1 and Roc1 expression plasmids. After 24 h, cells were lysed and immunoprecipitation against FLAG-tag or control IgG was performed. Western blots for the different SCF components are shown. (**b**) RNAi-mediated knockdown of SMART revealed by quantitative RT–PCR (qRT–PCR). Adult TA muscles were transfected with bicistronic expressing vectors that encode either oligo 4 or scramble and GFP. Two weeks later TA muscles were collected, RNA extracted and endogenous *SMART, MUSA1, Atrogin1, MuRF1* and *Fbxo31* expression were analysed by qRT–PCR, *n*=4. (**c**) Inhibition of SMART prevents muscle atrophy in denervated muscles. Adult muscle fibres were co-transfected with bicistronic expressing vectors that encode shRNAs against *SMART* (oligo 4) or scramble and GFP and denervated. Two weeks later cross-sectional area of transfected fibres, identified by GFP fluorescence, was measured. *n*=6 muscles for each group. (**d**) Densitometric quantification of polyubiquitinated proteins in muscle extracts transfected with shRNAi against *SMART* or scramble. Values are normalized to GAPDH and expressed as fold increase of fed control mice. *n*=3 muscles for each group. Data are shown as mean±s.e.m. Error bars indicate s.e.m. *P<0.05, **P<0.01 (Student's *t*-test). con, control; den, denervated; IB, immunoblotting.

IP experiments confirmed that SMART forms an SCF complex with Skp1, Cullin1 and Roc1 (Fig. 8a) and therefore belongs to the SCF family of E3 ligases. To confirm the role of SMART in promoting atrophy during denervation, we knocked down SMART in TA *in vivo*. Four different short hairpin RNAs (shRNAs) have been tested to specifically reduce SMART protein levels (Supplementary Fig. 18a), three of which efficiently knocked down SMART. Next, we transfected oligo 4 into innervated and denervated muscles. These shRNAs did not affect the expression of the other atrophy-related ubiquitin ligases, MUSA, MuRF1 and atrogin1 both at protein and mRNA level (Supplementary Fig. 18b, Fig. 8b). Importantly, SMART inhibition significantly protected denervated muscles from atrophy (Fig. 8c). This sparing is due to the fact that by blocking SMART we greatly reduced protein ubiquitination in denervated muscles (Fig. 8d, Supplementary Fig. 18c). Therefore, we have identified *SMART* as an additional critical gene whose upregulation is required for atrophy, yet must be carefully controlled to avoid excessive protein breakdown. In conclusion, our findings underline the concept that FoxO members are the master regulatory factor for protein homeostasis during catabolic conditions.

## Discussion

FoxOs are involved in a variety of biological process such as autophagy, apoptosis, ROS detoxification, glucose metabolism, DNA repair, cell cycle, stem cell maintenance and longevity^19^,^20^. Our work and that of others has shown that muscle atrophy is regulated by a transcription-dependent process that requires the expression of atrogenes. The transcription factors that orchestrate this complex gene network are still largely unknown but our findings highlight the FoxO family as one of the most important regulators. The finding that different genes involved in different pathways, including several ubiquitin ligases and proteasome subunits, are under FoxO regulation, is an important step towards the understanding of FoxO-dependent adaptation to stress such as nutritional deprivation. For instance, one of the most important adaptive responses that is induced to maintain cellular survival under stress conditions is autophagy^21^. Our data from different knockout mice highlight the concept that FoxOs are required to sustain autophagic flux under low nutrients and dominate mTOR signalling on autophagy regulation. It is worth underlining that FoxO deletion does not affect basal autophagic flux and indeed the knockout mice do not show any overt pathological phenotype that may resemble the features of muscle-specific Atg7 knockout mice^22^. This is in contrast with the phenotype of TSC1 knockout mice, which display hyperactivation of the mTORC1 pathway leading to inhibition of basal autophagic flux and resulting in a myopathic phenotype^23^. Altogether these findings suggest that basal autophagy is controlled by mTOR and not by FoxOs, while induction and maintenance of high autophagic flux for hours/days is entirely dependent on the FoxO family. Indeed, deletion of FoxO both in a muscle-specific manner and in an inducible muscle-specific manner resulted in suppression of autophagy during fasting in face of mTOR inhibition. Importantly, the demonstration that deletion of a single FoxO member is not sufficient to prevent muscle loss and autophagy activation supports the conclusion that there is significant redundancy among family members.

Among the family members, FoxO3 deletion is less compensated by the other factors suggesting that FoxO3 is the most critical factor for the atrophy programme. Interestingly, the sparing of muscle mass in the single knockout is not only due to an effect on the classical ubiquitin ligase atrogin1 and MuRF1 but is due to FoxO action on a different set of ubiquitin ligases. Indeed we have identified a group of novel ubiquitin ligases that are regulated by FoxO. Among the novel E3s, we identified a gene that encodes an F-box protein (FbxO21) whose function was completely unknown and that we named SMART. Gain- and loss-of-function experiments showed that FoxOs are required for SMART regulation and ChIP experiments revealed that FoxO1 and FoxO3 are recruited on the promoter. However, deletion of FoxO3 and not of FoxO1 completely blunted *SMART* induction after denervation, suggesting that *SMART* is mainly under FoxO3 regulation. However, when we overexpressed FoxO3 only *MUSA1* was induced, suggesting that FoxO3 is required for *SMART* induction but not sufficient and that other transcription factors are therefore involved in its regulation.

MUSA1 is a novel ubiquitin ligase that we found to be critical for muscle atrophy during denervation and fasting. Indeed, RNAinterference (RNAi)-mediated inhibition of MUSA1 expression, *in vivo,* in denervated muscles, spares muscle mass. Conversely, excessive MUSA1 induction exacerbates muscle loss causing muscle cachexia^16^. Expression of MUSA1 is regulated by Smad transcription factors^16^ in denervated muscles. The possibility that Smads and FoxO cooperate to promote MUSA1 expression is consistent with the finding that Smads require FoxO for the regulation of specific target genes. Therefore, it is possible that Smads are the partners of FoxO and both are required for the optimal activation of the atrogenes, at least during denervation. However, during fasting most of the atrophy-related genes are completely dependent on FoxO. In addition to the FoxO-dependent regulation of different ubiquitin ligases, it is important to emphasize that several proteasome subunits, ubiquitin C and the de-ubiquitinating enzyme USP14 are also controlled by FoxOs. These genes are critical in several steps of ubiquitination and proteasome-dependent degradation and might have an important role in control of protein degradation. For instance, it has recently been reported that expression of PSMD11 in human stem cells is sufficient to increase proteasome assembly and activity and, in *C. elegans*, a PSMD11 homologue induces resistance to oxidative stress and poly-glutamine aggregation and extends lifespan^24^.

Further work is needed to understand the interplay between the autophagy–lysosome and ubiquitin–proteasome systems in the context of different catabolic or even anabolic conditions, however, this work sets out the fundamentals for understanding the regulation of proteostasis in striated muscles.

## Methods

### Generation of muscle-specific FOXO1,3,4 knockout mice

Mice bearing *FoxO1,3,4*-floxed alleles (*FoxO1,3,4*^*f/f*^) 25 were crossed with transgenic mice expressing Cre either under the control of a Myosin Light Chain 1 fast promoter (*MLC1f-Cre*)^2^,^26^ or with transgenic expressing a *Cre-ER* driven by human skeletal actin promoter^27^. Genomic DNA isolated from *FoxO1,3,4*^*f/f*^ mice was subjected to PCR analysis. Cre-mediated recombination was confirmed by PCR with genomic DNA from gastrocnemius muscles using the primers Cre forward: 3′-CACCAGCCAGCTATCAACTCG-5′ and Cre reverse: 3′-TTACATTGGTCCAGCCACCAG-5′. Tamoxifen-inducible Cre was activated by special tamoxifen diet (*Tam400/Cre-ER Harlan*) or by i.p. Tamoxifen injection.

The genotyping analysis for each specific FoxO member was performed with the combination of the following primers: FoxO1 forward: 3′-GCT TAG AGC AGA GAT GTT CTC ACA TT-5′, FoxO1 reverse1: 3′-CCA GAG TCT TTG TAT CAG GCA AAT AA-5′, FoxO1 reverse2: 3′-CAA GTC CAT TAA TTC AGC ACA TTG A-5′, FoxO3 forward1: 3′-AGA TTT ATG TTC CCA CTT GCT TCC T-5′, FoxO3 forward2: 3′-TGC TTT GAT ACT ATT CCA CAA ACC C-5′, FoxO3 reverse: 3′-ATT CCT TTG GAA ATC AAC AAA ACT-5′, FoxO4 forward1: 3′- TGA GAA GCC ATT GAA GAT CAG A-5′, FoxO4 forward2: 3′-CTA CTT CAA GGA CAA GGG TGA CAG-5′, FoxO4 reverse: 3′-CTT CTC TGT GGG AAT AAA TGT TTG G-5′.

### Animals and *in vivo* transfection experiments

Animals were handled by specialized personnel under the control of inspectors of the Veterinary Service of the Local Sanitary Service (ASL 16—Padova), the local officers of the Ministry of Health. Mice were housed in individual cages in an environmentally controlled room (23 °C, 12-h light–dark cycle) with *ad libitum* access to food and water. All procedures are specified in the projects approved by the Italian Ministero Salute, Ufficio VI (authorization numbers C65) and by the Ethics Committee of the University of Padova. All experiments were performed on 2- to 4-month-old male (28–32*g*) and female mice (25–28*g*); mice of the same sex and age were used for each individual experiment. *In vivo* transfection experiments were performed by i.m. injection of expression plasmids in TA muscle followed by electroporation^8^. For fasting experiments, control animals were fed *ad libitum*; food pellets were removed from the cages of the fasted animals. Denervation was performed by cutting the sciatic nerve of the left limb, while the right limb was used as control. Muscles were removed at various time periods after transfection and frozen in liquid nitrogen for subsequent analyses.

### Gene expression analyses

Total RNA was prepared from TA muscles using Promega SV Total RNA Isolation kit. Complementary DNA (cDNA) generated with Invitrogen SuperScript III Reverse Transcriptase was analysed by quantitative real-time RT–PCR using Qiagen QuantiTect SYBR Green PCR Kit. All data were normalized to GAPDH and actin expression. The oligonucleotide primers used are shown in Supplementary Table 1.

### Plasmids and antibodies for Western blot

*In vivo* transfection experiments used the yellow fluorescent protein (YFP)–LC3^2^ and Fbxo21SMART-V5 plasmids. For the cloning of mouse *Fbxo21/SMART* gene, muscle cDNA was amplified by PCR using the primers forward: 3′-ACCATGGCGTCGGTAGCGGGGGACA-5′ and reverse: 3′-CTCGGCTGTGTCCTCCTTTGCACTG-5′.

The amplified sequence was cloned into pcDNA3.1/V5-His TOPO TA (Invitrogen) expression vector and sequenced. The list of the antibodies is described in Supplementary Table 2.

Uncropped blots are shown in Supplementary Fig. 19.

### *In vivo* RNAi

*In vivo* RNAi experiments were performed using at least three different sequences for each gene(Invitrogen BLOCK-iTTM Pol II miR RNAi Selected). The sequences are shown in Supplementary Table 3. For the validation of shRNA constructs, MEF cells were maintained in DMEM/10% FBS and transfected with shRNA constructs using Lipofectamine 2000 (Invitrogen). Cells were lysed 24 h or 48 h later and immunoblotting was performed.

### Gene expression profiling

For each of the 4 conditions (*FoxO1,3,4*^*f/f*^ fed or starved and *FoxO1,3,4*^*−/−*^ fed or starved), we collected the gastrocnemius muscles of three mice thus yielding six muscles per condition.

RNA was prepared from these muscles using the TRIzol method (Life Technologies) followed by cleanup with the RNeasy kit (Qiagen). RNA concentration was determined by spectrophotometry and quality of the RNA was monitored using the Agilent 2100 Bioanalyzer (Agilent Technologies). RNA of the six muscles per condition was pooled equimolarly and used for further microarray analysis. cRNA was prepared, labelled and hybridized to Affymetrix Mouse Genome 430 2.0 Arrays using Affymetrix-supplied kits and according to standard Affymetrix protocols. Expression values were summarized using the Mas 5.0 algorithm. Genes that were up or downregulated on starvation compared with the fed condition were determined using Excel software. A threshold of 1.5 was used for the fold up or downregulation consistent with the fold change that can be reliably detected with these type of arrays.

### *In vivo* ChIP assay

We performed ChIP assay in adult skeletal muscles using the Magna ChIP A/G Chromatin Immunoprecipitation Kit (Millipore)^2^,^28^. Soluble chromatin was co-immunoprecipitated with rabbit polyclonal anti-FKHRL1 (FoxO3) sc-11351X (Santa Cruz Biotechnology), rabbit polyclonal anti-FKHR (FoxO1) sc-11350X (Santa Cruz Biotechnology) or an equal amount of normal rabbit IgG, sc-2027 (Santa Cruz Biotechnology). After decrosslinking of the DNA, samples were subjected to quantitative RT–PCR. The oligonucleotide primers used are shown in Supplementary Table 4. The regions of amplification contain the FOXO-binding sites for the promoter studied.

### Immunoblotting and IP

Frozen gastrocnemius muscles were powdered by pestle and mortar and lysed in a buffer containing 50 mM Tris pH 7.5, 150 mM NaCl, 5 mM MgCl_2_, 1 mM DTT, 10% glycerol, 2% SDS, 1% Triton X-100, Roche Complete Protease Inhibitor Cocktail, 1 mM PMSF, 1 mM NaVO_3_, 5 mM NaF and 3 mM β-glycerophosphate. The lysis buffer used for MEF and C2C12 cells contained 50 mM Tris pH 7.5, 150 mM NaCl, 5 mM MgCl_2_, 1 mM DTT, 10% glycerol, 1 mM EDTA, 0.5% Triton X-100 and the protease inhibitors listed above. Alternatively, lysis buffer contained 50 mM Tris HCl pH 7.2, 250 mM NaCl, 2% NP40, 0.1% SDS, 0.5% sodium deoxycholate 2.5 mM, EDTA pH8 with anti-phosphatase and anti-protease. The samples were immunoblotted and visualized with SuperSignal West Pico Chemiluminescent substrate (Pierce). Blots were stripped using Restore Western Blotting Stripping Buffer (Pierce) according to the manufacturer's instructions and reprobed if necessary. For Myosin analysis, gastrocnemius muscles were homogenized in myosin extraction buffer containing 1 M Tris pH 6.8, 10% SDS and 80% glycerol. SDS–PAGE was performed using polyacrylamide gel with a high glycerol concentration, which allows the separation of MYH isoforms. Myosins were identified with Coomassie blu staining.

For co-IP experiment C2C12 muscle cell lines were transfected with V5-SMART, HA-Skp1, FLAG-Cul1 and FLAG-Roc1 expression plasmids. After 24 h, cells were lysed in a buffer containing 50 mM Tris pH 7.5, 100 mM NaCl, 5 mM MgCl2, 1 mM DTT, 0.5% Triton X-100, protease and phosphatase inhibitors. About 1 mg of total protein was incubated at a ratio of 1:100 with the mouse monoclonal anti-FLAG antibody or non specific mouse IgG along with 30 μl of Protein A/G PLUS-Agarose sc-2003 (Santa Cruz Biotechnology) overnight at 4 °C. The beads were then washed three times with PBS plus Roche Complete Protease Inhibitor Cocktail 1X and were finally resuspended in 30 μl LDS Sample Buffer 1 × (NuPAGE Life Technolohy) and 50 mM DTT to be further analysed by Western blotting.

### Histology and microscopy

Cryosections of transfected TA muscles were examined in a fluorescence microscope Leica DM5000B equipped with a Leica DFC300-FX digital charge-coupled device camera by using Leica DC Viewer software. Cryosections of TA were stained for haematoxilin and eosin and as well as for Succinate dehydrogenase and Periodic acid-Schiff. Cross-sectional area was measured using ImageJ software in at least 400 transfected fibres and compared with the area of age-matched control.. The fibre diameter was calculated as caliper width, perpendicular to the longest chord of each myofibre. The total myofibre number was calculated from entire muscle section based on assembled mosaic image ( × 20 magnification). Fibre typing was determined by immunofluorescence using combinations of the following monoclonal antibodies: BA-D5 that recognizes type 1 MyHC isoform and SC-71 for type 2A MyHC isoform. Images were captured using a Leica DFC300-FX digital charge-coupled device camera by using Leica DC Viewer software, and morphometric analyses were made using the software ImageJ 1.47 version.

### LC3-vesicle quantification

Cryosections of fed and 30-h fasted muscles from control and *FoxO1,3,4*^*−/−*^ mice that were transfected *in vivo* with YFP–LC3 were examined using an epifluorescence Leica DM5000B microscope equipped with a Leica DFC300-FX digital charge-coupled device camera by using Leica DC Viewer software. The fluorescent dots were counted and normalized for cross-sectional area.

### Autophagic flux quantification

We monitored autophagic flux in 30 h of starvation using colchicine^15^. Briefly MLC *FoxO1,3,4*^*−/−*^ and *FoxO1,3,4*^*f/f*^ mice were treated with 0,4 mg kg^−1^ colchicine or vehicle by i.p. injection and starved. The treatment was repeated at 15 h prior to muscle harvesting.

### Adenovirus production and myotube transfection

*FOXO3* was cloned into a shuttle vector pAdTrack-CMV, which contains green fluorescent protein (GFP) under the control of a separate promoter. C2C12 mouse myoblasts were cultured in DMEM 10% FCS until the cells reached confluence. The medium was then replaced with DMEM 2% horse serum (‘differentiation medium') and incubated for 4 days to induce myotube formation before proceeding with experiments. For infection, myotubes were incubated with adenovirus at a multiplicity-of-infection of 250 in differentiation medium for 18 h, and then the medium was replaced. The infection efficiency was typically >90%.

### Muscle physiology

To determine force and contraction kinetics of gastrocnemius, mice were anesthetized by a mixture of Xylor and Zoletil, and a small incision was made from the knee to the hip, exposing the sciatic nerve. Before branching of the sciatic nerve, TeflonTM-coated 7 multistranded steel wires (AS 632; Cooner Sales, Chatsworth, CA, USA) were implanted with sutures on either sides of the sciatic nerve. To avoid recruitment of the ankle dorsal flexors the common peroneal nerve was cut. Torque production of the stimulated plantar flexors was measured using a muscle lever system (Model 305C; Aurora Scientific, Aurora, ON, Canada).

*Ex vivo* force measurements on soleus muscles were performed by dissecting it from tendon to tendon under a stereomicroscope and subsequently mounting between a force transducer (KG Scientific Instruments, Heidelberg, Germany) and a micromanipulator-controlled shaft in a small chamber, in which oxygenated Krebs solution was continuously circulated and temperature was maintained at 25 °C. The stimulation conditions were optimized, and the length of the muscle was increased until force development during tetanus was maximal.

Data were analysed using the PowerLab system (4SP, ADInstruments) and software (Chart 4, ADInstruments). The sciatic nerves were stimulated using supramaximal square wave pulses of 0.1-ms duration. Force generation capacity was evaluated by measuring the absolute maximal force that was generated during isometric contractions in response to electrical stimulation (frequency of 75–150 Hz, train of stimulation of 500 ms). Maximal isometric force was determined at L0 (length at which maximal tension was obtained during the tetanus). Force was normalized to the muscle mass as an estimate of specific force. Following force measurements, animals were killed by cervical dislocation and muscles were dissected, weighed and frozen in liquid nitrogen or in isopentane precooled in liquid nitrogen.

### Statistical analysis and general experimental design

The sample size was calculated using size power analysis methods for *a priori* determination based on the s.d., and the effect size was previously obtained using the experimental methods employed in the study. For animal studies, we estimated sample size from expected number of knockout mice and littermate controls, which was based on mendelian ratios. We calculated the minimal sample size for each group by at least four organisms. Considering a likely drop-off effect of 10%, we set sample size of each group at five mice. To reduce the s.d., we minimized physiological variation by using homogenous animals with same sex and same age. The exclusion criteria for animals were pre-established. In case of death, cannibalism or sickness, the animal was excluded from analysis. Tissue samples were excluded in cases such as cryo-artefacts, histological artefacts or failed RNA extraction. We included animals from different breeding cages by random allocation to the different experimental groups. Animal experiments were not blinded, however, when applicable, the experimenters were blinded to the nature of samples by using number codes until final data analysis was performed. Statistical tests were used as described in the figure legends and were applied on verification of the test assumptions (for example, normality). Generally, data were analysed by two-tailed Student's *t*-test. For all graphs, data are represented as means±s.e.m. For the measurement variables used to compare KO animals versus controls, or innervated animals versus denervated ones, the variance was similar between the groups.

## Author contributions

G.M. generated the triple FoxO1,3,4 muscle-specific knockout. G.M., V.R. and F.P. performed biochemical analyses, morphological, immunohistochemical and RNA analysis, muscle transfections and mouse treatments. G.M. and A.A. performed *in vivo* ChIP assay. A.A. performed immunohistochemical analysis. L.F. performed protein analysis and immunohistochemical analysis. A.S. performed immunohistochemical analysis and RNA analysis. J.Z. performed Adeno-mediated FoxO3 overexpression in C2C12 myotubes, RNA extraction and qRT–PCR. R.A. performed microarray analysis. B.B. analysed muscle mechanics. R.A.D. provided FoxO floxed mice. G.M., V.R., F.P., A.L.G. and M.S. were involved in data analysis. G.M, V.R and M.S. designed the study, analysed the data and wrote the manuscript. All authors discussed the results and corrected the manuscript.

## Additional information

**Accession codes:** The gene expression profiling data discussed in this publication have been deposited in the NCBI Gene Expression Omnibus and are accessible through GEO Series accession number GSE52667.

**How to cite this article:** Milan, G. *et al.* Regulation of autophagy and the ubiquitin–proteasome system by the FoxO transcriptional network during muscle atrophy. *Nat. Commun.* 6:6670 doi: 10.1038/ncomms7670 (2015).

## Supplementary Material

Supplementary Information Supplementary Figures 1-19, Supplementary Tables 1-4
