# Supplementary material for: Regulation of autophagy and the ubiquitin–proteasome system by the FoxO transcriptional network during muscle atrophy
Source: Nat Commun. 2015 Apr 10;6:6670. doi: 10.1038/ncomms7670 (PMC4403316; doi:10.1038/ncomms7670)
Supplement: Supplementary Information — Supplementary Figures 1-19, Supplementary Tables 1-4 [file ncomms7670-s1.pdf]

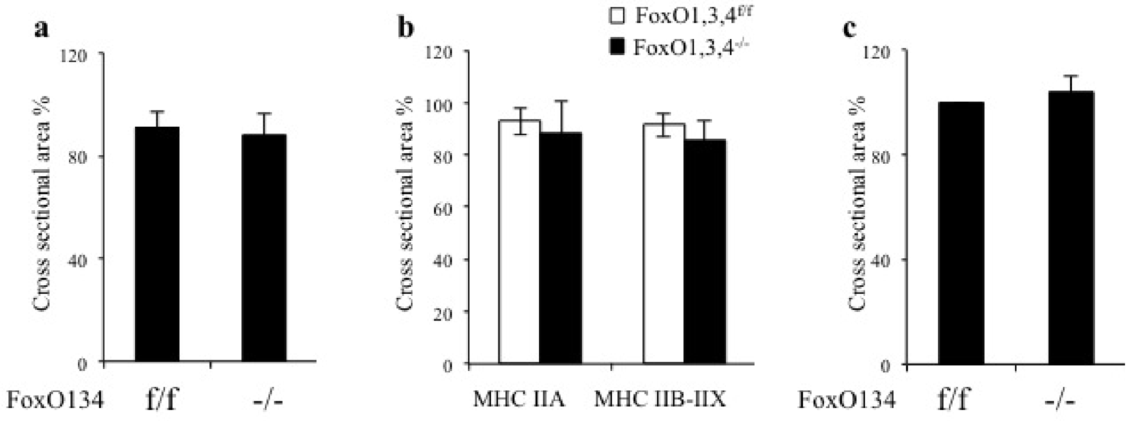

Supplementary Fig. 1 Average fiber size of fast glycolytic muscles did not differ from controls **(a)** Cross-sectional area (CSA) of *Tibialis Anterior* (TA) muscles from FoxO1,3,4 knockout (-/-) and control (f/f) mice. **(b)** Cryosections of TA from *FoxO1,3,4$^{f/f}$* and *FoxO1,3,4$^{-/-}$* mice were immunostained for dystrophin and for the fast myosins type IIA (MHCIIA), type IIB (MHCIIB) or type IIX (MHCIIX). Size of individual fibers was determined. **(c)** Cross-sectional area of *Gatrocnemius* muscles from *FoxO1,3,4$^{-/-}$* and control mice. Values are shown as means ± s.e.m. of data from 4 muscles in each group. Error bars indicate s.e.m.

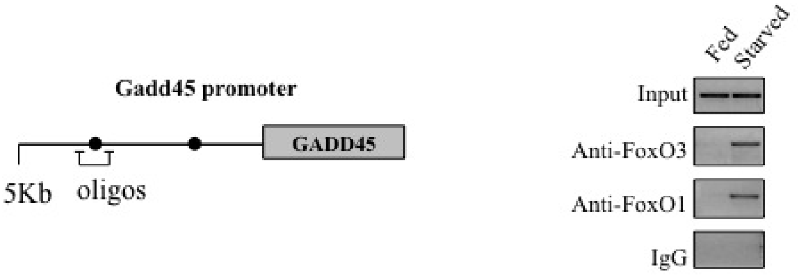

Supplementary Fig. 2 Starvation induces FoxOs recruitment on target promoter Chromatin Immunoprecipitation (ChIP) PCR showing that FoxO1 and FoxO3 translocate to the nucleus and bind to FoxO binding element of *GADD45*α promoter, a bona fide FoxO targetin starved muscles. IgG is a negative control.

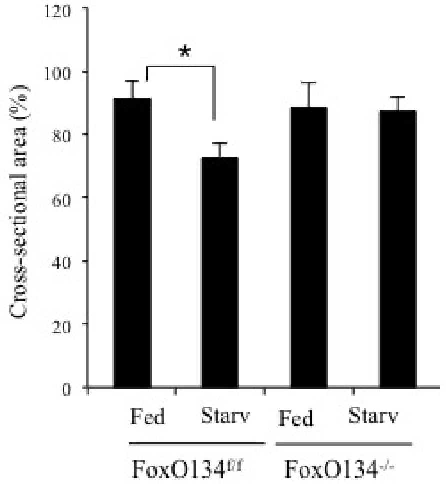

Supplementary Fig. 3 Deletion of FoxOs prevents atrophy during starvation The graph shows the cross-sectional area of myofibers of TA from *FoxO1,3,4*$^{f/f}$ and *FoxO1,3,4*$^{-/-}$ mice, in fed and starved condition. Values are shown as means ± s.e.m. of data from 4 muscles in each group. Error bars indicate s.e.m. *p<0.05(Student's *t*-test). Starv: starved.

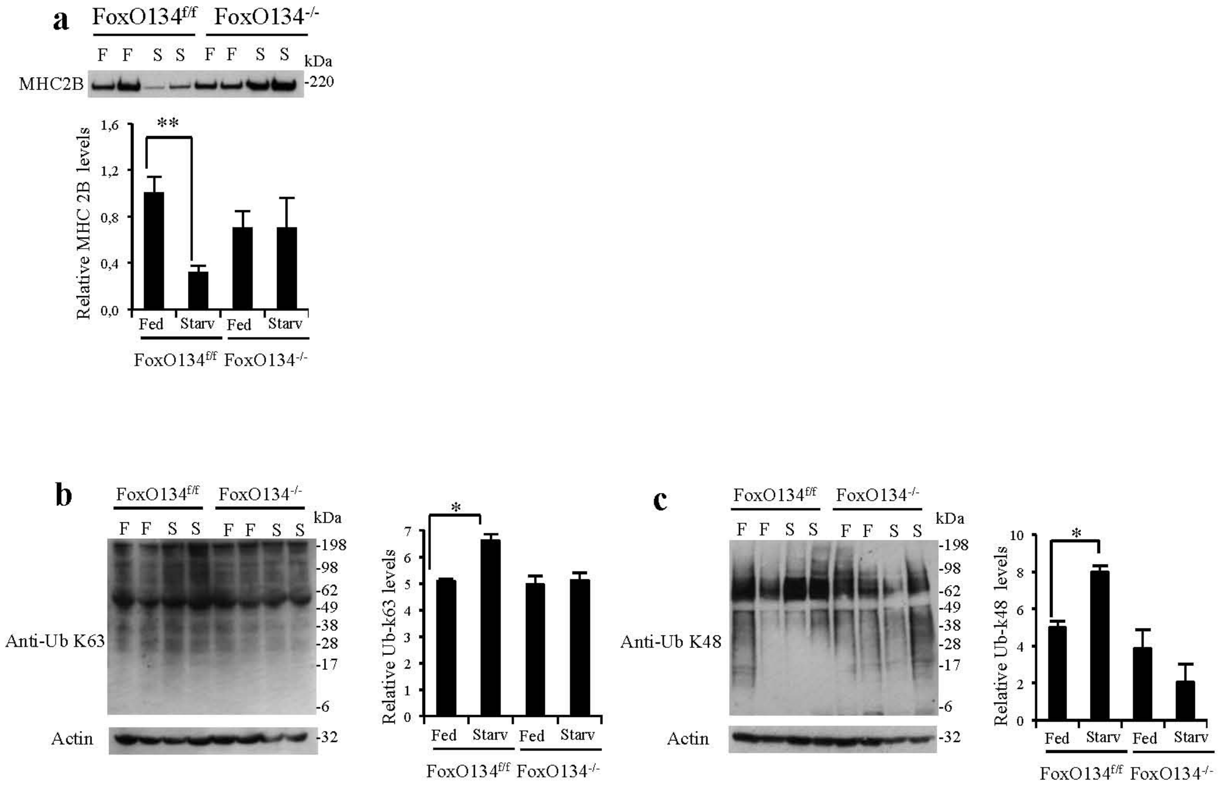

Supplementary Fig. 4 FoxO inhibition prevents protein ubiquitination during starvation

(a) Representative Immunoblot of MCHIIB in fed and in starved muscles. Quantification of MHCIIB content is shown in the graph. n=4 muscles in each group. **(b, c)** Protein extracts from control and starved muscles of *FoxO1,3,4*$^{-/-}$ and *FoxO1,3,4*$^{f/f}$ mice were immunoblotted against **(b)** K63-polyUbiquitin and **(c)** K48-polyUbiquitin chains. The graphs show densitometry quantification of two different experiments of n=4 for each group. Data are mean ± s.e.m. Error bars indicate s.e.m. *p<0.05 **p<0.01 (Student's *t*-test).F: fed, S: starved, Starv: starved

,

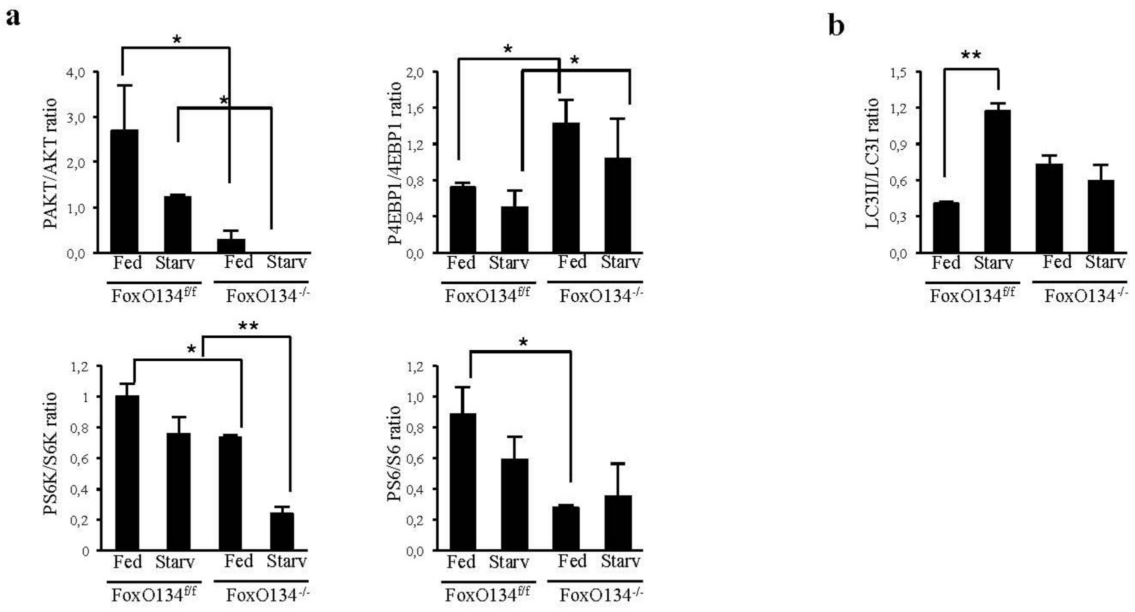

Supplementary Fig. 5 Akt pathway and lipidation of LC3 are affected by FoxO1,3,4 deletion(a) Densitometric quantification of western blots of p-AKT, p-4EBP1, p-S6K1 and p-S6. n=4 muscles in each group. **(b)** Densitometric quantification of LC3II band in control and FoxOs knockout mice. The data show that LC3 lipidation occurs in $FoxO1,3,4^{f/f}$ but not in $FoxO1,3,4^{-/-}$. n=4 muscles in each group. Values are mean ± s.e.m. Error bars indicate s.e.m. *p<0.05, **p<0.01(Student's *t*-test) Starv: starved

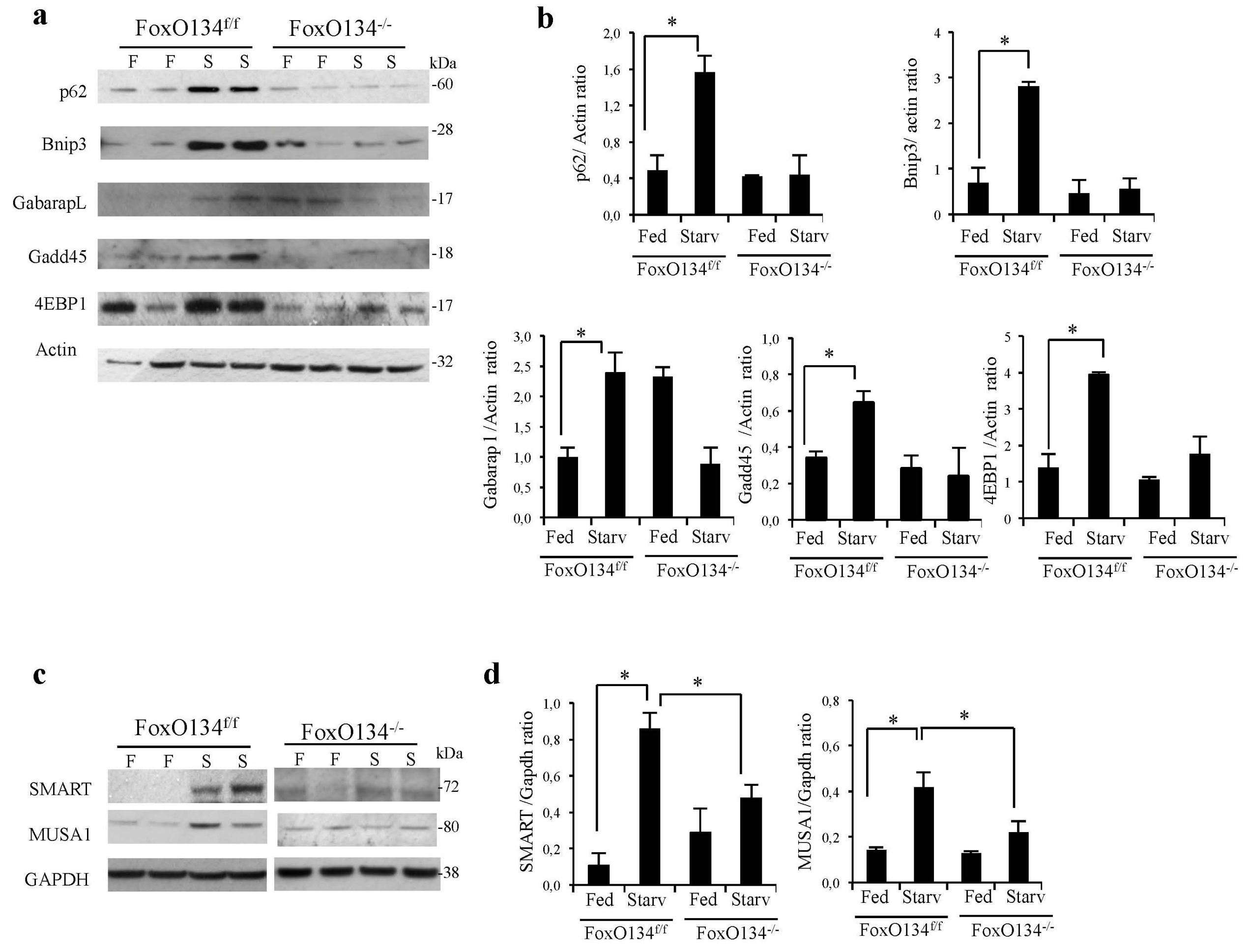

Supplementary Fig. 6 FoxOs are required for the expression of several atrogenes

(a) Representative Western blots of several atrophy-related genes. Protein extracts from fed and starved muscles of *FoxO1,3,4*$^{f/f}$ and *FoxO1,3,4*$^{-/-}$ mice were immunoblotted against p62, Bnip3, Gabarapl, Gadd45α and 4EBP1. Blots are representative of two groups of experiments. **(b)** The graphs show the densitometric quantification of the blots. At least 4 muscles for each group were used. **(c)** Representative Immunoblots of the novel atrophy-related ubiquitin ligase SMART and MUSA1. **(d)** Densitometric quantification was performed from 3 fed *gastrocnemius* from *FoxO1,3,4*$^{f/f}$ and from *FoxO1,3,4*$^{-/-}$ and 4 starved *gastrocnemius* from *FoxO1,3,4*$^{f/f}$ and from *FoxO1,3,4*$^{-/-}$. Data are shown as mean ± s.e.m. Error bars indicate s.e.m. *$p<0.05$ (Student's *t*-test). F: fed, S: starved, Starv: starved.

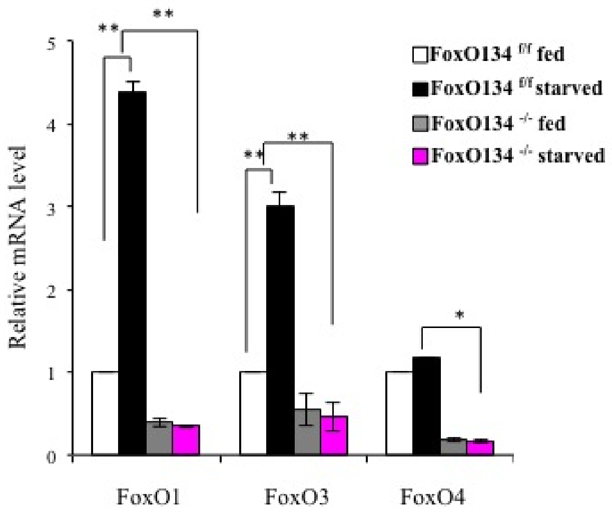

Supplementary Fig. 7 Tamoxifen-inducible muscle-specific FoxO1,3,4 knockout mice *FoxO1*, *FoxO3*, *FoxO4* mRNA expression were quantified by RT PCR in TA muscles of *FoxO1,3,4*$^{-/-}$ and control mice after tamoxifen treatment. Values are mean ± s.e.m. Error bars indicate s.e.m.*p<0.05, **p<0.01(Student's *t*-test)

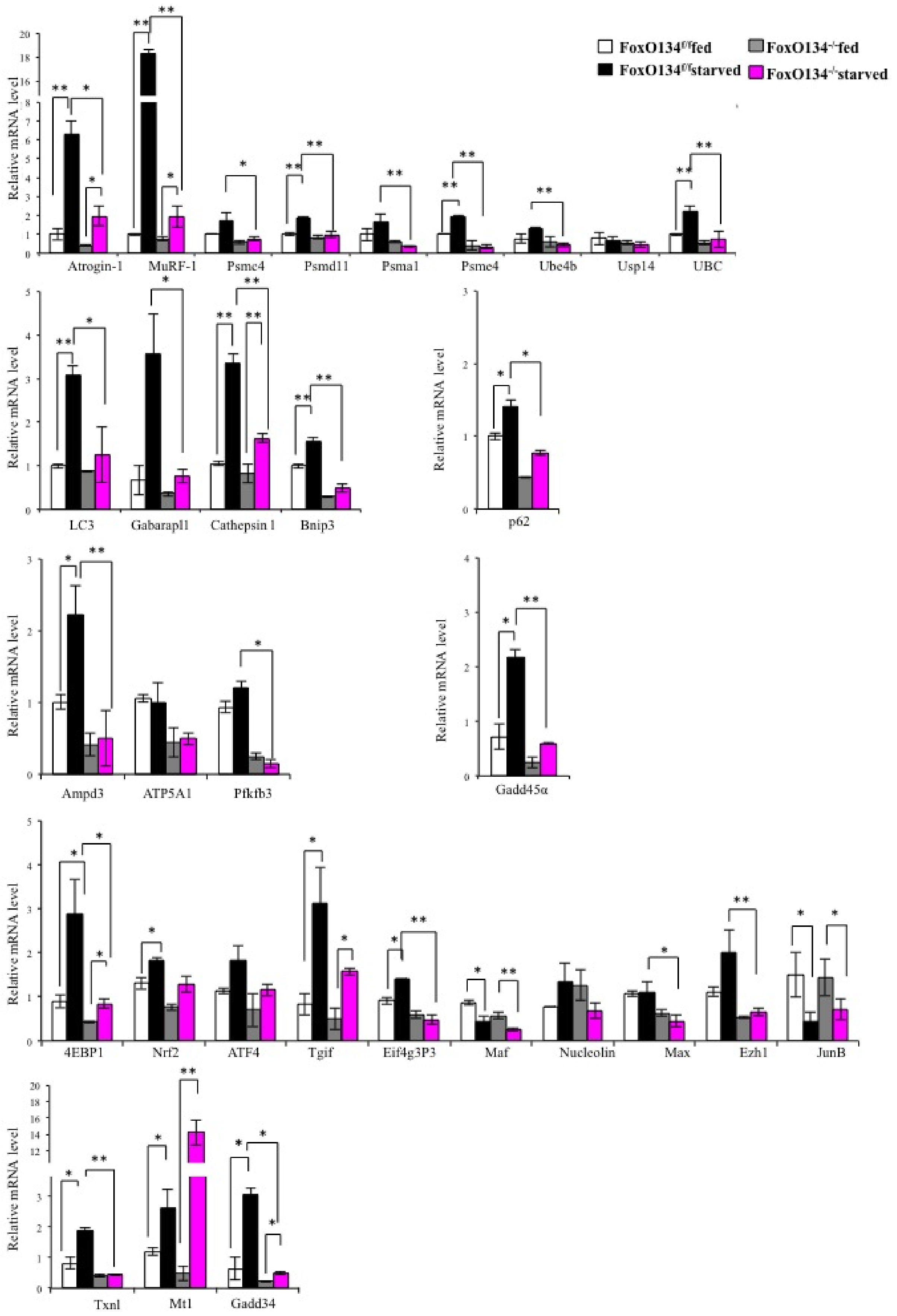

Supplementary Fig. 8 Acute inhibition of FoxOs prevents atrogenes induction Quantitative RT-PCR of atrogenes from fed and 24 hours starved TA of control and tamoxifen-induced *FoxO1,3,4*$^{-/-}$ mice. Data are normalized to *GAPDH* and expressed as fold increase of control fed animals. Values are mean ± s.e.m. *p<0,05,**p<0,01. Error bars indicate s.e.m. (Student's *t*-test) n=4 muscles in each group.

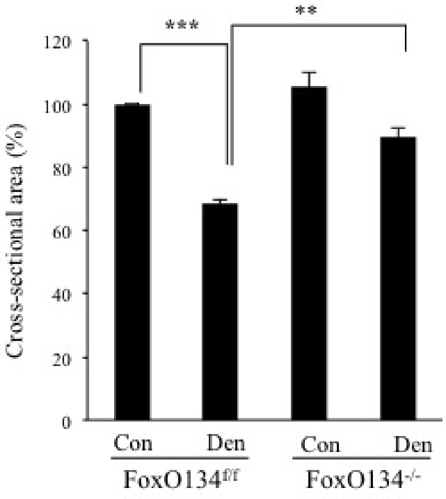

Supplementary Fig. 9 Ablation of FoxOs partially prevents denervation-dependent atrophy The graph shows the cross-sectional area of myofibers on innervated and denervated TA of *FoxO1,3,4*$^{f/f}$ and *FoxO1,3,4*$^{-/-}$mice. Values are shown as means ± s.e.m. of data from 4 muscles in each group. Error bars indicate s.e.m. *p<0.05, **p<0.01(Student's *t*-test). Con: control, Den: denervated.

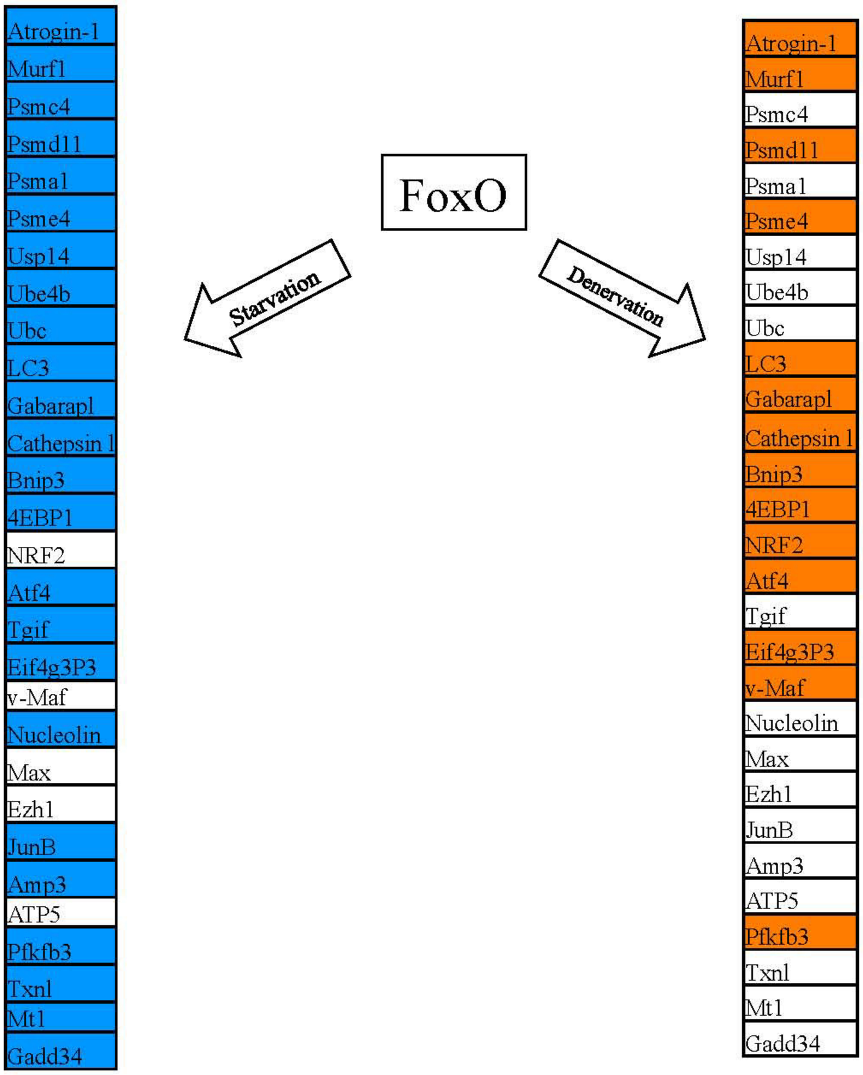

Supplementary Fig. 10 FoxO-dependent atrogenes during starvation and denervation. Schematic representation showing the genes found to be FoxO-dependent based on microarray analysis. Different colours indicate FoxO-dependent genes that have been validated by RT-PCR in fasting (blue) and in denervation (orange).

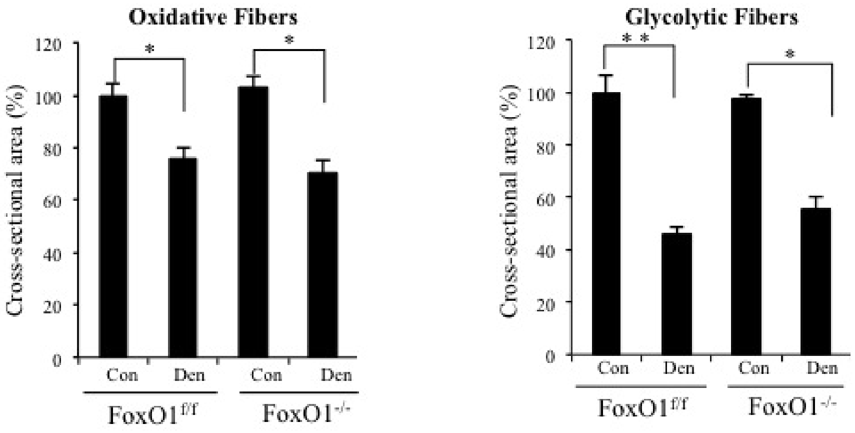

Supplementary Fig. 11 Lack of FoxO1 does not protect from denervation atrophy CSA of TA muscles from knockout and control mice. Bar graphs represent the CSA of oxidative (left) and glycolytic (right) fibers, revealed by SDH staining. Values are mean ± s.e.m. of data from 4 muscles in each group. Error bars indicate s.e.m. *p<0.05, **p<0.01(Student's *t*-test). Con: control, Den: denervated.

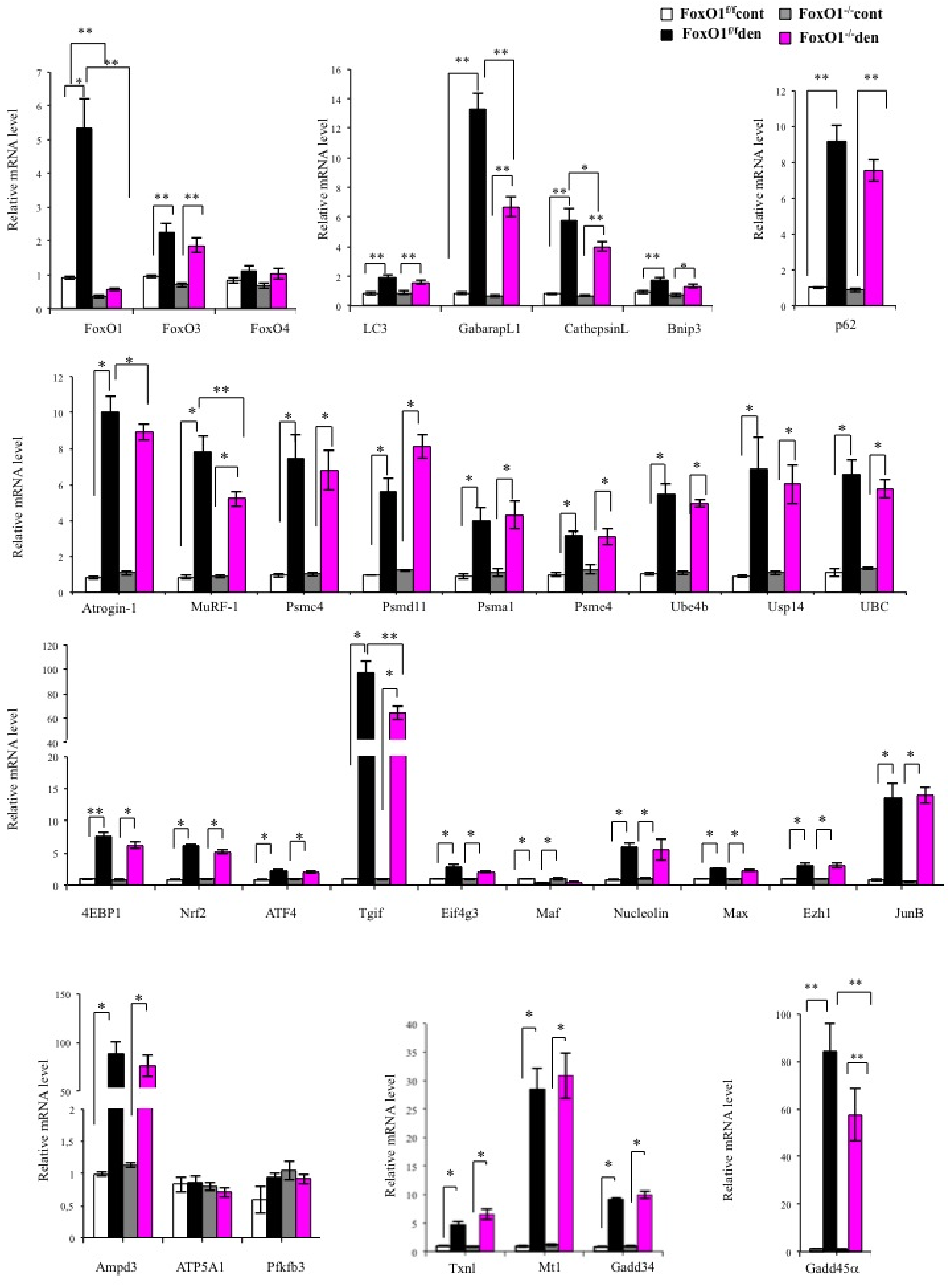

Supplementary Fig. 12 FoxO1 is required for optimal induction of few atrophy-related genes Quantitative RT-PCR of the atrogenes after 3 days from denervation. Data are normalized to *GAPDH* and expressed as fold increase of control innervated muscles. Values are mean ± s.e.m. Error bars indicate s.e.m.*p<0.05, **p<0.01(Student's *t*-test) Cont: control, Den: denervated

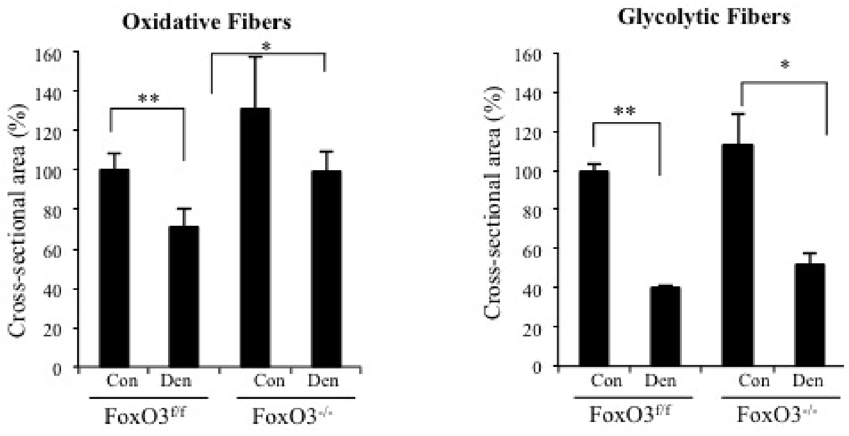

Supplementary Fig. 13 FoxO3 deletion partially protects from atrophy after denervation. CSA of oxidative (left) and glycolytic (right) fibers of TA muscles, revealed by SDH staining from knockout and control mice . Values are mean ± s.e.m. of data from 4 muscles in each group. Error bars indicate s.e.m. *p<0.05, **p<0.01(Student's *t*-test) Con: control, Den: denervated.

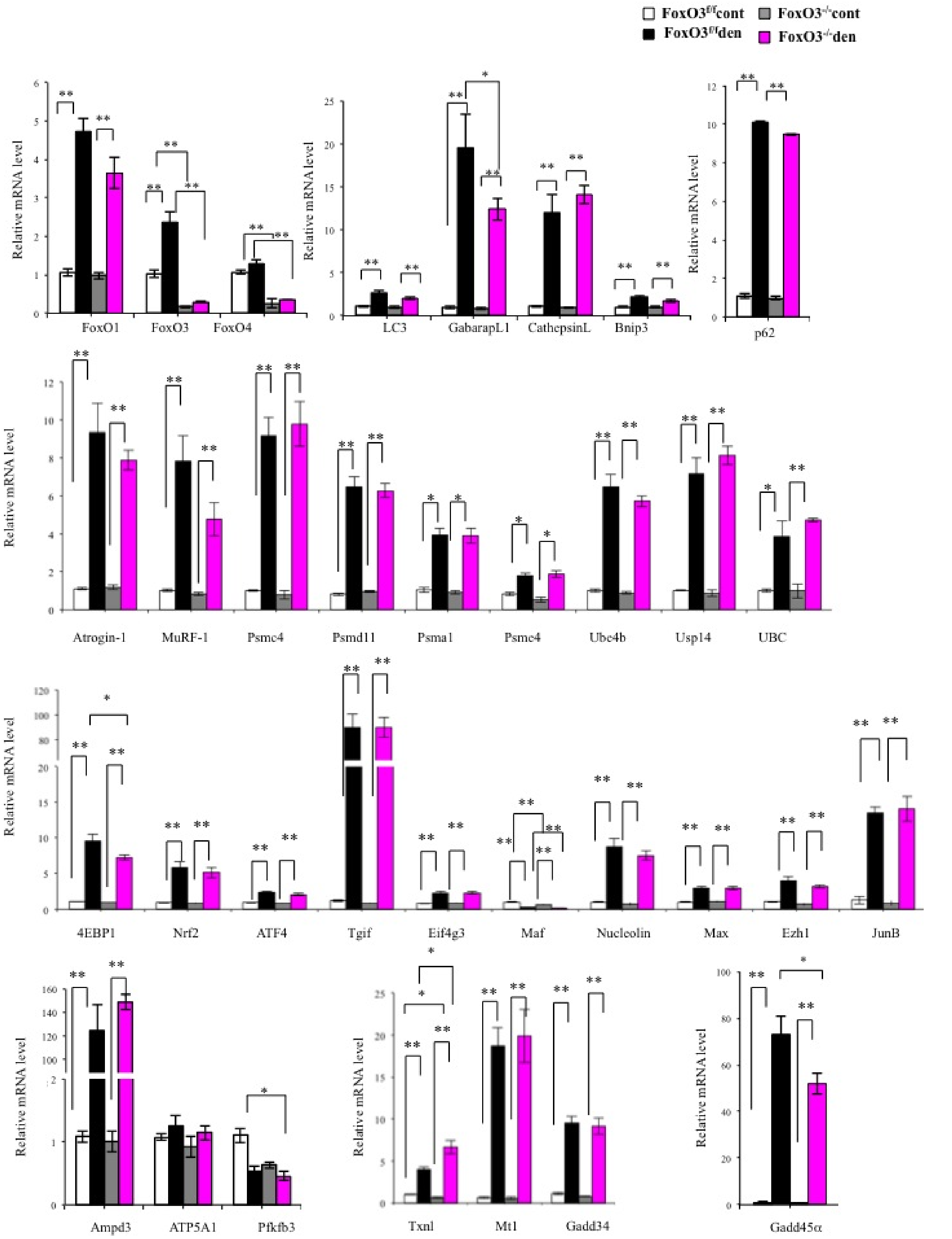

Supplementary Fig 14 FoxO3 is required for the induction of few atrogenes in denervation Quantitative RT-PCR of the indicated atrogenes after 3 days from denervation. Data are normalized to *GAPDH* and expressed as fold increase of control innervated muscles. Values are mean ± s.e.m. Error bars indicate s.e.m. *$p<0.05$, **$p<0.01$(Student's *t*-test).cont: control, den: denervated.

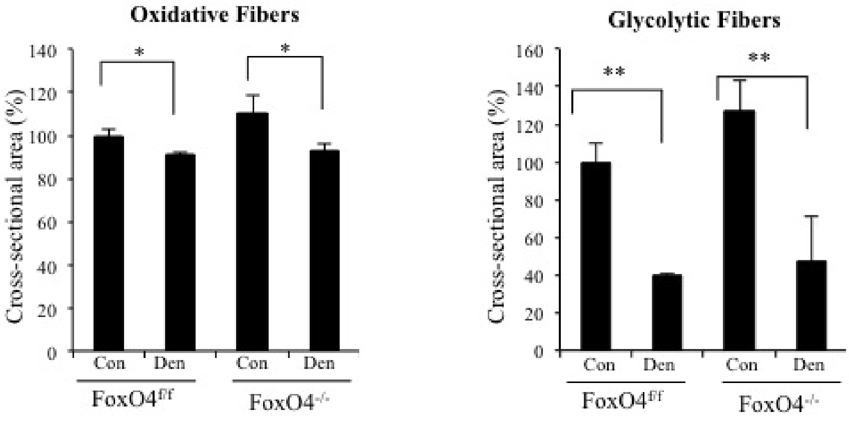

Supplementary Fig. 15 Ablation of FoxO4 does not prevent muscle atrophy after denervation. CSA of oxidative (left) and glycolytic (right) fibers of TA muscles, revealed by SDH staining from knockout and control mice. Values are mean ± s.e.m. of data from 4 muscles in each group. Error bars indicate s.e.m. *p<0.05, **p<0.01(Student's *t*-test). Con: control, Den: denervated.

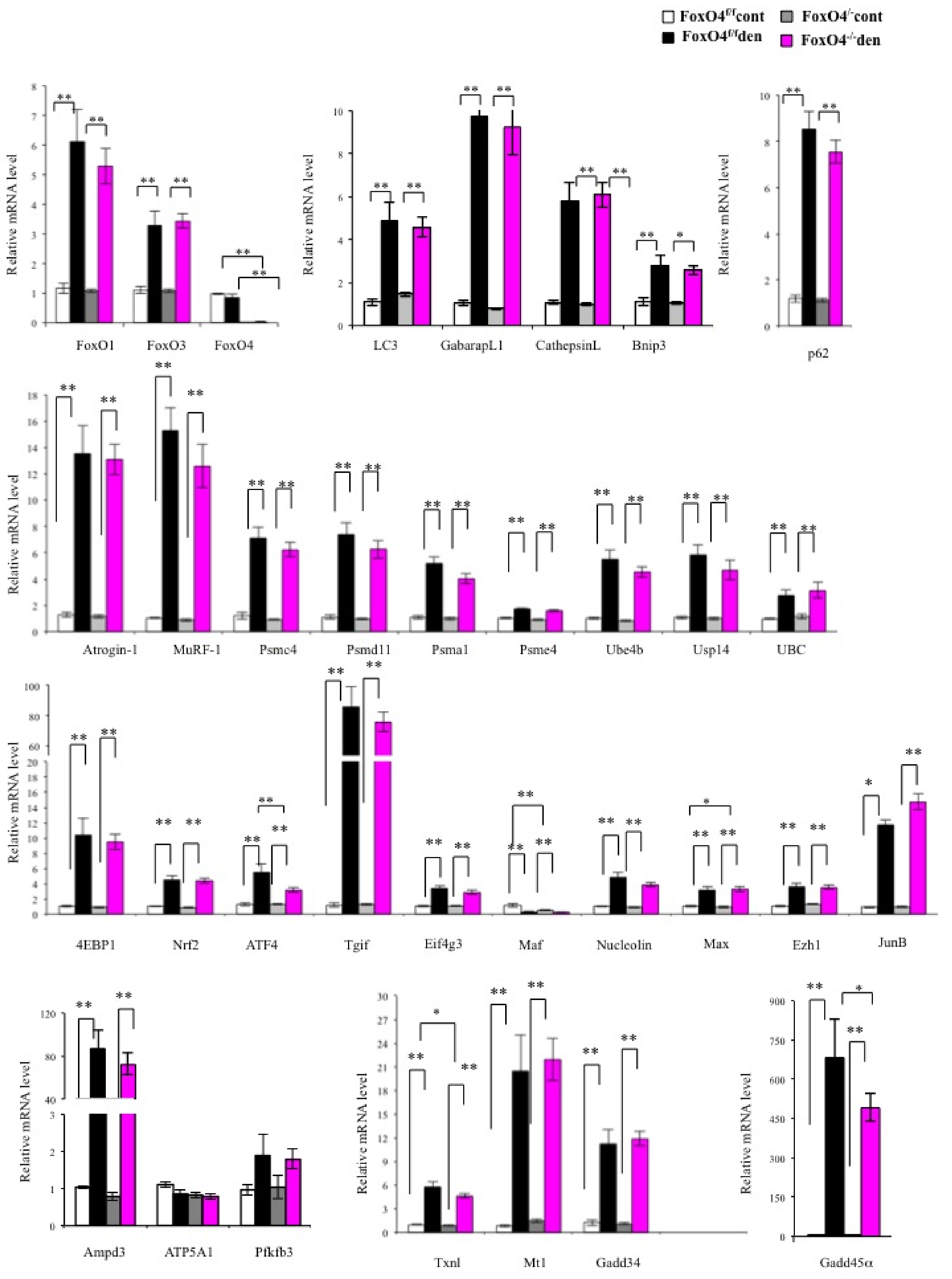

Supplementary Fig. 16 FoxO4 is required for optimal induction of Gadd45α. Quantitative RT-PCR of the indicated atrogenes after 3 days from denervation. Data are normalized to *GAPDH* and expressed as fold increase of control innervated muscles. Values are mean ± s.e.m. Error bars indicate s.e.m. *p<0.05, **p<0.01(Student's *t*-test) . cont: control, den: denervated.

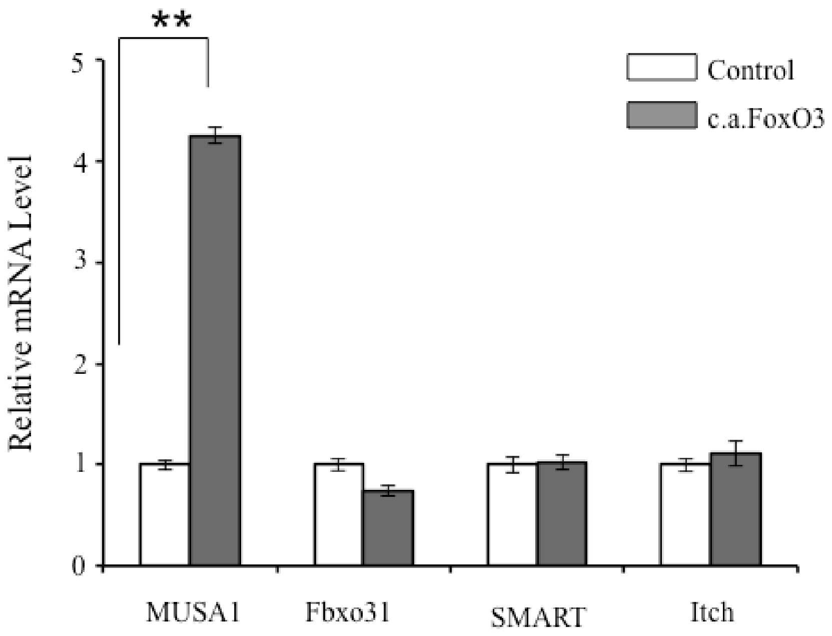

Supplementary Fig 15 FoxO3 is sufficient to induce MUSA1, but not SMART, Itch or Fbox31. Quantitative RT-PCR of the different ubiquitin ligases in myotubes overexpressing c.a.FoxO3 and controls. Values are means ± s.e.m.Error bars indicate s.e.m**p<0.01(Student's *t*-test) c.a: constitutively active.

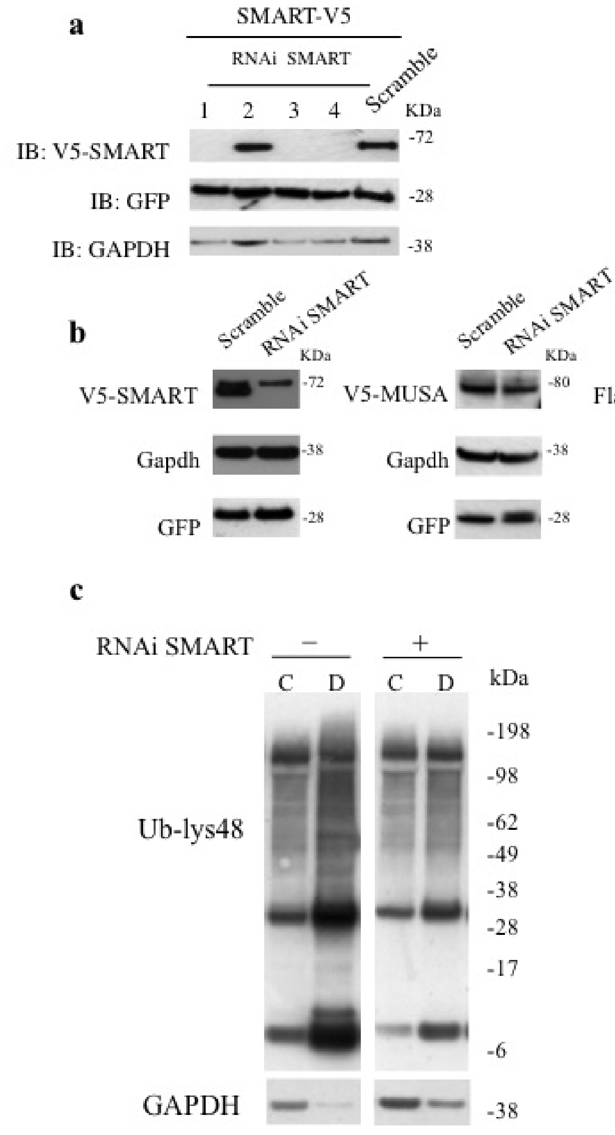

Supplementary Fig 18 RNAi-mediated knockdown of SMART. (a) RNAi-mediated knockdown of SMART revealed by immunoblotting. Murine embryonic fibroblasts (MEFs) were transfected with vectors expressing different shRNAs against *SMART* together with vectors encoding murine V5-SMART. IB: immunoblotting **(b)** The shRNA against *SMART* does not interfere with MUSA, Atrogin1, and MuRF1 expression. C2C12 myoblast were co-transfected with vectors expressing shRNA against *SMART* (oligo 4) or scramble together with vectors encoding V5- SMART, V5-MUSA, Flag-MURF1 or Myc-Atrogin1. After 48 hours proteins were extracted and immunoblotted for V5, Flag or Myc tag. GADPH and GFP were detected as control of loading and transfection efficiency respectively. **(c)** Representative immunoblots against K48-polyUbiquitin chains of innervated or denervated TA muscles that were transfected with shRNAs against SMART or scramble. C: control, D: denervated.

Fig. 2d

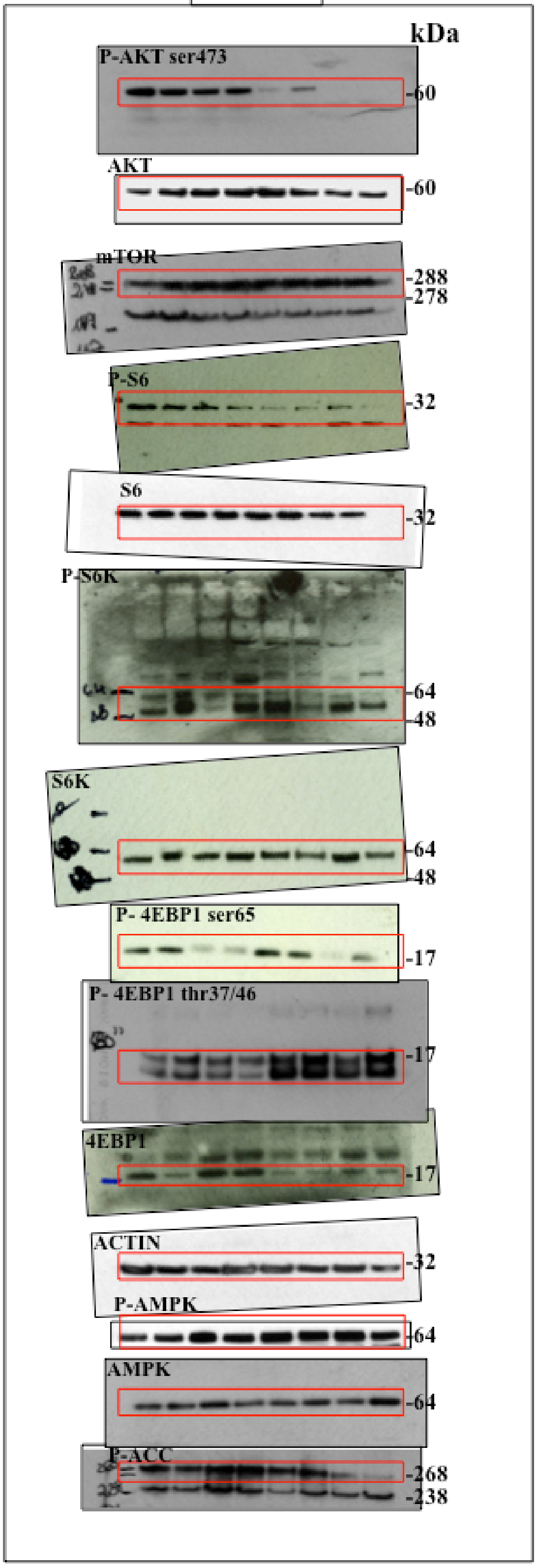

Fig. 2e

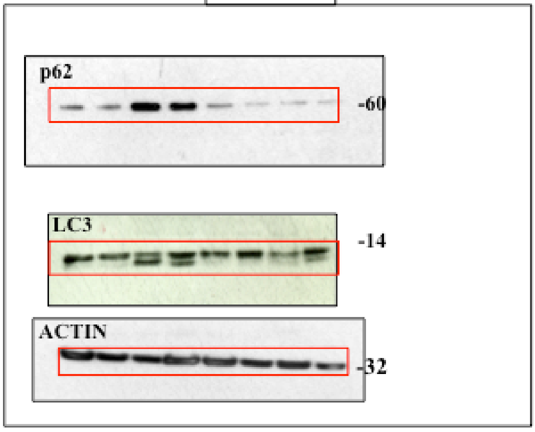

Fig. 2g

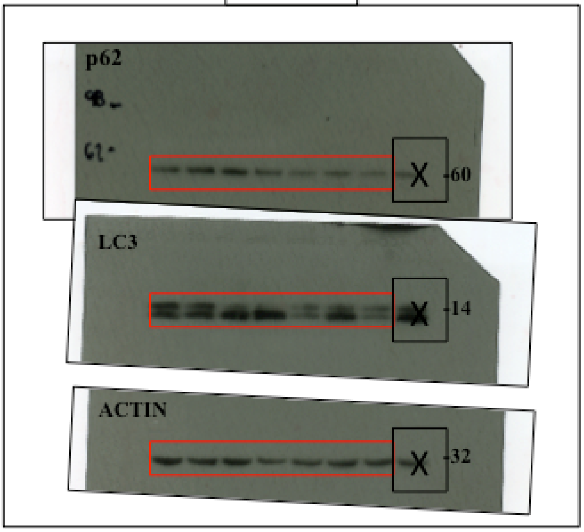

Fig. 4c

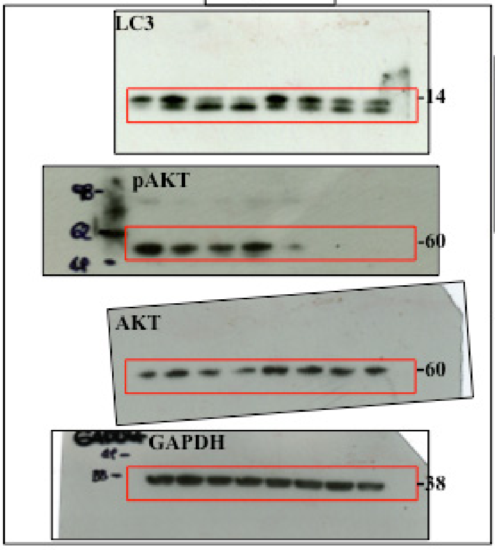

Fig. 4d

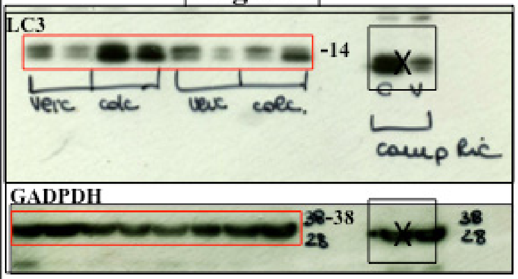

Fig. 5d

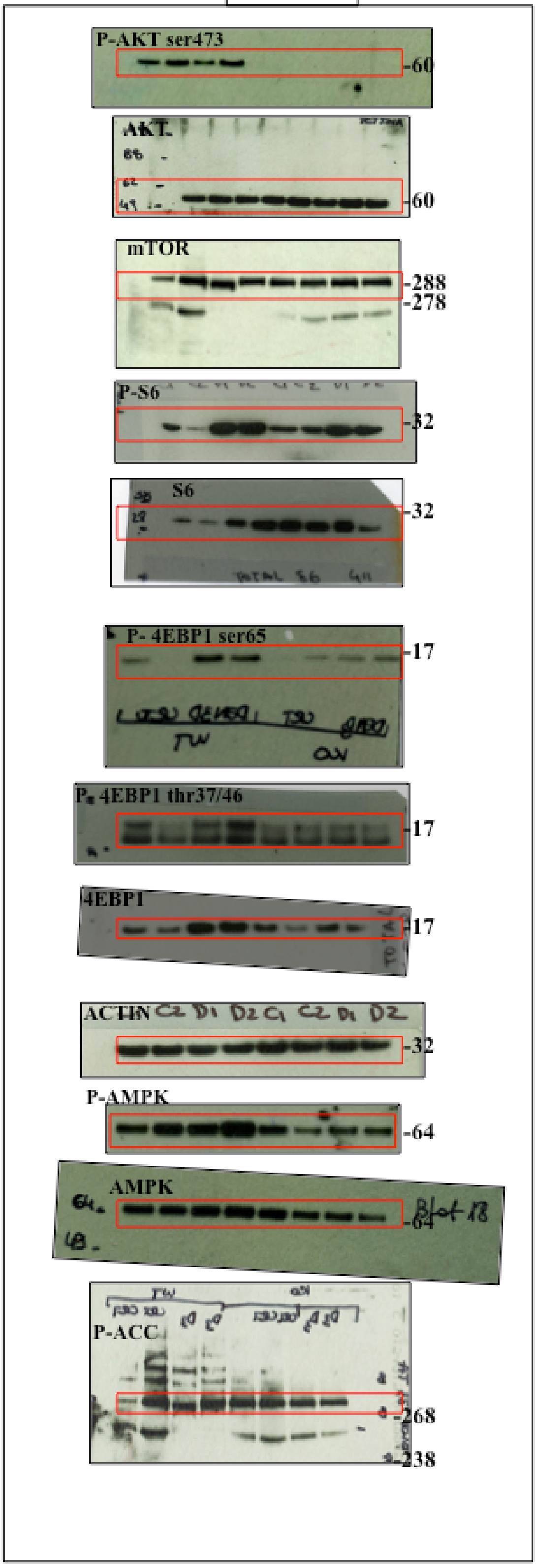

Fig. 5e

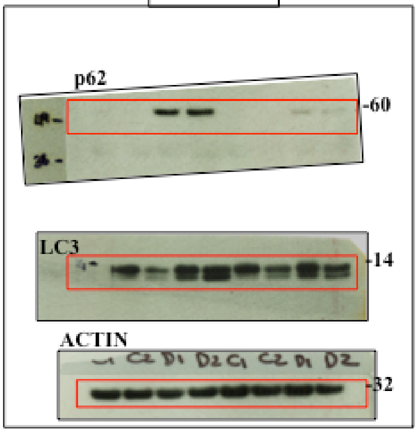

Supplementary Fig 19. Uncropped scans of blots.

Supplementary Table 1 Primers used for Quantitative PCR Analyses

Gene	Forward primer (5'-3')	Reverse Primer (5'-3')
4EBP1	CCTCCTTGTGCCTCTGTCTA	GCCTAAGGAAAGATGGGTGT
Actin	CTGGCTCCTAGCACCATGAAGAT	GGTGGACAGTGAGGCCAGGAT
Ampd3	GCGGAGAAGGTGTTTGCTA	CAGTCTTGTTGTGTTGGCATC
ATF4	TCCTGAACAGCGAAGTGTTG	ACCCATGAGGTTTCAAGTGC
ATP5A1	GAGAGAGCAGCCAAGATGAAC	GACACGGGACACAGACAAAC
Atrogin-1	GCAAACACTGCCACATTCTCTC	CTTGAGGGGAAAGTGAGACG
Bnip3	TTCCACTAGCACCTTCTGATGA	GAACACCGCATTTACAGAACAA
CathepsinL	GTGGACTGTTCTCACGCTCAAG	TCCGTCCTTCGCTTCATAGG
Eif4g3P3	GAAACGGAGAAGAGGCTGAG	GATACAGGCGGGCATAAACT
Ezh1	GTGTTTTCTTTTCTTCTTCTGTGC	GTGTTTTCTTTTCTTCTTCTGTGC
SMART	TCAATAACCTCAAGGCGTTC	GTTTTGCACACAAGCTCCA
FbxO31	GTATGGCGTTTGTGAGAACC	AGCCCCAAAATGTGTCTGTA
FoxO1	GCTGGGTGTCAGGCTAAGAG	GAGGGGTGAAGGGCATCT
FoxO3	CGCTGTGTGCCCTACTTCA	CCCGTGCCTTCATTCTGA
FoxO4	CGGAGTGAAAGGGACAGTTTAG	CCCTGTGGCTGACTTCTTATTC
GabarapL	CATCGTGGAGAAGGCTCCTA	ATACAGCTGGCCCATGGTAG
Gadd34	AGAGAAGACCAAGGGACGTG	CAGCAAGGAATGGACTGTG
Gadd45 a	GAAAGTCGCTACATGGATCAGT	AAACTTCAGTGCAATTTGGTTC
GAPDH	CACCATCTTCCAGGAGCGAG	CCTTCTCCATGGTGGTGAAGAC
Itch	CCACCCACCCCACGAAGACC	CTAGGGCCCGAGCCTCCAGA
JunB	GATCCCTATCGGGGTCTCAA	GAGGCTAGCTTCAGAGATGC
LC3b	CACTGCTCTGTCTTGTGTAGGTTG	TCGTTGTGCCTTTATTAGTGCATC
Maf	GCATCATCAGCCAGTGCGGC	AGGTGCGCCTTCTGTTCGCT
Max	CGAAAACGTAGGGACCACAT	GATCTTGCCTTCTCCAGTGC
Mt1	GCCTGCAAGAACTGCAAGTG	CCTTTGCAGACACAGCCCT
MUSA1	TCGTGGAATGGTAATCTTGC	CCTCCCGTTTCTCTATCACG
MuRF-1	ACCTGCTGGTGGAAAACATC	ACCTGCTGGTGGAAAACATC
Nrf2	CTCTGACTCTGGCATTTCACTG	ACACTTCAGGGGCACTATCTA
Nucleolin	TTTATCAAAGTGCCCCAGAA	GTTCTGCCCTCAATTTCCAT
p62	CCCAGTGTCTTGGCATTCTT	AGGGAAAGCAGAGGAAGCTC
Pfkfb3	GCCTCTTGACCCTGATAAATGT	TCTTGCCTCTGCTGGACA
Psma1	CATTGGAATCGTTGGTAAAGAC	GTTCATCGGCTTTTTCTGC
Psmc4	AGGACGAGCAGAAGAACCTG	AATAGTTAGAGCCTGTGGTGGAG

Supplementary Table 1 Primers used for Quantitative PCR Analyses

Gene	Forward primer (5'-3')	Reverse Primer (5'-3')
Psmd11	GAGTTCCAGAGAGCCCAGTC	AACCCAGTTCAAGGATGCTC
Psme4	TTGTAGATGCATGCCGACTC	ACCTGGGTGAGTTTTGGTTC
Tgif	TTTCCTCATCAGCAGCCTCT	CTTTGCCATCCTTTCTCAGC
Txnl	GGTGGGAGTGAAGCCGGTCG	CGGGGCAATCCGAAGACACG
UBC	CGTCGAGCCCAGTGTTACCACC	ACCTCCCCCATCACACCCAAGA
Ube4b	TGTCATCTTCCTTTCTTCTCTCTC	TGGATTTTCATCTCGTGTCTG
Usp14	CACGAGTTGCTTCGTATTCC	TTCAGGGTTCCTCCTTTCAC

Supplementary Table 2 Antibodies used in this study.

Antibody	Customer	Dilution	Analysis
rabbit anti-dystrophin	Abcam ab 15277	1:100	IF
Mouse anti SC-71-s	DSHB	1:100	IF
Mouse anti BF-F3	DSHB	1:100	IF
Mouse anti BA-D5	DSHB	1:100	IF
rabbit anti- actin	Abcam A4700	1:10000	WB
rabbit anti-total Akt	Cell Signaling #9272	1:1000	WB
rabbit anti-phospho-Akt (Ser473)	Cell Signaling #3787	1:1000	WB
rabbit anti-phospho-S6K (Thr 389)	Cell Signaling #9205	1:1000	WB
rabbit anti-total S6K	Cell Signaling #9202	1:1000	WB
rabbit anti-phospho-S6	Cell Signaling #2215	1:1000	WB
rabbit anti-total S6	Cell Signaling #2217	1:1000	WB
rabbit anti-LC3	Sigma L7543	1:1000	WB
rabbit anti-P62	Sigma P0067	1:1000	WB
rabbit anti-phospho-AMPK(Thr 172)	Cell Signaling #2535	1:1000	WB
rabbit anti-total AMPK	Cell Signaling #2532	1:1000	WB
rabbit anti-phospho-4EBP1 (Ser 65)	Cell Signaling #9455	1:1000	WB
rabbit anti-phospho-4EBP1 (Thr 37/46)	Cell Signaling #9459	1:1000	WB
rabbit anti-total 4EBP1	Cell Signaling #9452	1:1000	WB
rabbit anti-Gabarap	Santa Cruz Biotechnology sc-28938	1:1000	WB
rabbit anti-Gadd45α	Santa Cruz Biotechnology sc-797	1:1000	WB
rabbit anti-GFP	Santa Cruz Biotechnology sc- 8334	1:1000	WB
rabbit anti-PACC	Cell Signaling #3661	1:1000	WB
rabbit anti-mTOR	Cell Signaling #2983	1:1000	WB
mouse anti-V5	Life Technologies	1:1000	WB
mouse anti-Fbxo21	Abcam 119762	1:1000	WB
mouse anti-GAPDH	Abcam ab 8245	1:5000	WB
mouse anti-HA	Sigma H3663	1:1000	WB
mouse anti-Flag	Sigma A222	1:1000	IP/WB
mouse IgG2A anti-puromycin	12D10	1:30000	WB
mouse anti-puromycin	Hybridoma Bank PMY-2A4	1:30000	WB
mouse- anti ubiquitinylated proteins (Fk2)	Millipore #04-263	1:1000	WB
mouse- anti ubiquitin antibody, Lysin 63 specific	Millipore #05-1308	1:1000	WB
mouse anti-β-tubulin	Sigma T8328	1:1000	WB

Supplementary Table 2 Antibodies used in this study.

Antibody	Customer	Dilution	Analysis
mouse IgG2A anti-puromycin	12D10	1:30000	WB
Rabbit anti-FoxO1	Cell signalling #9946	1:1000	WB
Rabbit anti-FoxO3	Cell signalling #9946	1:1000	WB
Goat anti Fbxo30	Santa Cruz Biotechnology	1:100	WB
Goat anti mouse IgG	Biorad 1706516	1:2000	WB
Goat anti rabbit IgG	Biorad 1706515	1:2000	WB
Goat anti mouse IgG-2b DyLight405	Jackson Immunoresearch 115-475-207	1:200	IF
Goat anti mouse IgG-1 DyLight405	Jackson Immunoresearch115-485-205	1:200	IF
Goat anti mouse IgG (H+L) Cy3	Jackson Immunoresearch 115-026-003	1:200	IF
Goat anti Rabbit IgG-(H+L) Cy3	Jackson Immunoresearch 111-166-003	1:200	IF

Supplementary Table 3 Oligos Used for siRNA production

mSMART(1)	top (5'-3')	TGCTGTGGAGAAGGAAGCTACAATCTGTTTTGGCCACTGACTGACAGATTGTATTCCTTCTCCA
	botton (5'-3')	CCTGTGGAGAAGGAATACAATCTGTCAGTCAGTGGCCAAAACAGATTGTAGCTTCCTTCTCCAC
mSMART(2)	top (5'-3')	TGCTGAAGACAAGACAGAAAGACTTCGTTTTGGCCACTGACTGACGAAGTCTTTGTCTTGTCTT
	botton (5'-3')	CCTGAAGACAAGACAAAGACTTCGTCAGTCAGTGGCCAAAACGAAGTCTTTCTGTCTTGTCTTC
mSMART(3)	top (5'-3')	TGCTGTTAGGATACACACCAGCTCATGTTTTGGCCACTGACTGACATGAGCTGGTGTATCCTAA
	botton (5'-3')	CCTGTTAGGATACACCAGCTCATGTCAGTCAGTGGCCAAAACATGAGCTGGTGTGTATCCTAAC
mSMART(4)	top (5'-3')	TGCTGTTTGCACACAAGCTCCACAATGTTTTGGCCACTGACTGACATTGTGGATTGTGTGCAAA
	botton (5'-3')	CCTGTTTGCACACAATCCACAATGTCAGTCAGTGGCCAAAACATTGTGGAGCTTGTGTGCAAAC

Supplementary Table 4 Primers Real Time used for ChIP- qPCR

	Forward primer (5'-3')	Reverse Primer (5'-3')
Atrogin-1 S1	GGACACAGTGCTTGATAGACAGTC	GTCTCTTCCTTGCTCACGTTTGTC
Atrogin-1 S2	CTGGCAGGGAGGAGCCTAATGAATC	GGGAGTGGCAAAGCCGTCTC
Atf4	AGAGCTTTGGCTAGGTGTCC	GGGGTAACTGTGGCGTTAGA
Bnip3	TGGGTCAGGTCACTAGAAGC	GCCCTCGTATAACCTTAGCA
Cathepsinl	CCCAGCGAGGACAATTCAGAC	CGTGTCATCCTCTGCTGCA
LC3	CATGCCTTGGGACACCAGAT	ACCTTCTTCAAGTGCTGTTTGT
Gadd45alfa	CCTCCTTCCAACCATAATACTC	GGTGCCCTCTGTTTTTGA
MuRF-1	AAGCAGGTGCCACTCTCTGT	AGCTTCACACCTGTCCTTCG
Tgif1	GGCTCACAACCATCCGTAAC	GCTGGCTTCCTTTTGGTCTA
p62	TAGGCAGGTGCTCTGTCACT	GGCTGCATGAGGCTTCTAA
4EBP1	GTTCAAATCCCAGCAACCAC	CCCTCTACCACCTTCCCTCT
MUSA1 S1	TACTCTTGCTGGGCATTTTC	CCACATTTTGAGGGAGGTGT
MUSA1 S2	ACTCTAACCCCGCCTCAGTA	ACAAAATCCACAGGCAAATG
MUSA1 S3	GGAGCAGTTTTCCATTCTTG	ACCACACTTGCAGGACACAT
SMART	GCAAGTAACAAGGTCAAGCAGTACA	AACAGTGGTCAAAGGAGTGATCA
itch	GAACCATCCTCCCAACTCAGAA	ATGTGAAGATGCCATTGGTTACC
